# Multiplexed, targeted profiling of single-cell proteomes and transcriptomes in a single reaction

**DOI:** 10.1186/s13059-016-1045-6

**Published:** 2016-09-19

**Authors:** Alex S Genshaft, Shuqiang Li, Caroline J. Gallant, Spyros Darmanis, Sanjay M. Prakadan, Carly G. K. Ziegler, Martin Lundberg, Simon Fredriksson, Joyce Hong, Aviv Regev, Kenneth J. Livak, Ulf Landegren, Alex K. Shalek

**Affiliations:** 1 Institute for Medical Engineering & Science, Massachusetts Institute of Technology, Cambridge, MA USA; 2 Department of Chemistry, Massachusetts Institute of Technology, Cambridge, MA USA; 3 Broad Institute of MIT and Harvard, Cambridge, MA USA; 4 Ragon Institute of Massachusetts General Hospital, Massachusetts Institute of Technology, and Harvard University, Cambridge, MA USA; 5 Fluidigm Corporation, South San Francisco, CA USA; 6 Department of Immunology, Genetics & Pathology and Science for Life Laboratory, Uppsala University, Uppsala, Sweden; 7 Departments of Bioengineering and Applied Physics, Stanford University and Howard Hughes Medical Institute, Stanford, CA USA; 8 Division of Health Sciences & Technology, Harvard University and Massachusetts Institute of Technology, Cambridge, MA USA; 9 Olink Proteomics, Uppsala, Sweden; 10 Department of Electrical Engineering & Computer Science, Massachusetts Institute of Technology, Cambridge, MA USA; 11 Department of Biology and Koch Institute, MIT, Boston, MA 02142 USA; 12 Howard Hughes Medical Institute, Chevy Chase, MD 20815 USA

**Keywords:** Single-cell transcriptomics, Single-cell proteomics, Single-cell multi-omics, Proximity extension assay, Metadata

## Abstract

We present a scalable, integrated strategy for coupled protein and RNA detection from single cells. Our approach leverages the DNA polymerase activity of reverse transcriptase to simultaneously perform proximity extension assays and complementary DNA synthesis in the same reaction. Using the Fluidigm C1™ system, we profile the transcriptomic and proteomic response of a human breast adenocarcinoma cell line to a chemical perturbation, benchmarking against in situ hybridizations and immunofluorescence staining, as well as recombinant proteins, ERCC Spike-Ins, and population lysate dilutions. Through supervised and unsupervised analyses, we demonstrate synergies enabled by simultaneous measurement of single-cell protein and RNA abundances. Collectively, our generalizable approach highlights the potential for molecular metadata to inform highly-multiplexed single-cell analyses.

**Electronic supplementary material:**

The online version of this article (doi:10.1186/s13059-016-1045-6) contains supplementary material, which is available to authorized users.

## Background

Recently, there has been an explosion of papers that utilize highly-multiplexed single-cell RNA profiling (through quantitative reverse transcription-polymerase chain reaction (qRT-PCR) [1, 2] or sequencing [3–9]) to investigate the extent, causes, and consequences of cellular heterogeneity. Although incipient, this body of work has convincingly demonstrated that covariation in gene expression across single cells can be used to identify distinct cell states and circuits, as well as their molecular markers and drivers, respectively [1, 2, 4–10]. In parallel, orthogonal studies have shown that endogenous protein levels and activity can vary dramatically between single cells [1, 11–14] with important functional consequences and predictive power [1, 11, 12, 14]. Nevertheless, a gene’s RNA and protein levels do not necessarily correlate [15–18] and the long-standing question of how RNA expression patterns covary with and are driven by the levels and activities of various protein species remains underexplored [10, 15, 19, 20].

To date, given the limited number of RNAs and proteins that can be simultaneously assayed in situ and the noise associated with any one measurement [3, 10], the state-of-the-art has been to quantitatively record the levels of select cell surface proteins (index sort) during the fluorescence-activated cell sorting (FACS)-based isolation of single cells that normally precedes single-cell RNA profiling. This and related approaches can effectively link precision single-cell protein measurements – and thus much of the scientific community’s accrued data and knowledge – to high-dimensional single-cell RNA profiles, enabling deeper insights [1, 10, 21–24]. However, these techniques are fundamentally limited in both the number (n_total_ ~15 due to spectral overlap [10, 25]) and type of protein targets (extracellular, since the fixation and permeabilization required for intracellular staining can degrade cellular RNA [26, 27]) they can assay.

**Fig. 1:**
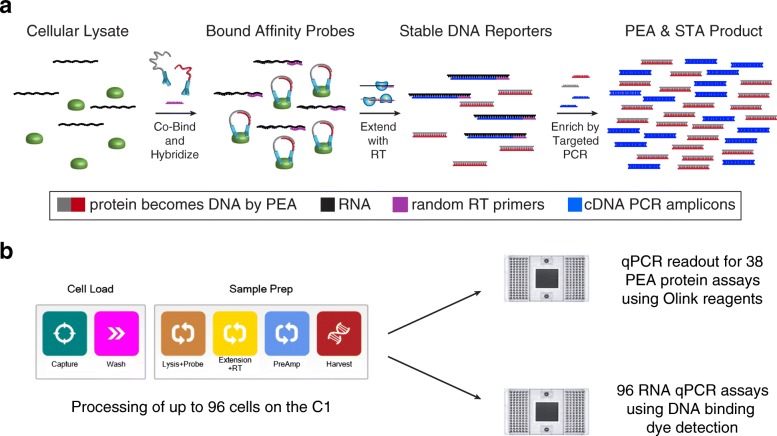
Overview of the integrated PEA/STA protocol. **a** *Workflow* for PEA/STA detection in single cells. *Gray* and *red* represent PEA probe specific and complementary oligonucleotides and their copies, *black* represents RNA, *purple* represents random primers, and *blue* represents cDNA reverse transcribed and copied from RNA. **b** *Schematic* of the script used on the C1 system to perform PEA/STA

One potential way to address these shortcomings of scope and scale is to encode the abundance of both RNAs and proteins in DNA space using reverse transcription (RT) [2] and proximity extension assays (PEA) [28], respectively – this renders both analytes stable, amplifiable, and quantitatively detectable (Fig. 1). The latter method, PEA, is a continuation of the proximity ligation assay (PLA) [29] that relies on the binding of two antibodies in proximity to generate a DNA reporter with low background noise. In PEA, pairs of monoclonal or polyclonal antibodies are functionalized with pairs of single-stranded DNA oligonucleotides with complementary 3’ ends. When co-localized by binding to their target protein, these oligonucleotides hybridize and can be extended by a DNA polymerase to generate a protein-indexed DNA molecule. This DNA reporter can then be co-amplified with complementary DNA (cDNA) [2] and co-detected by qPCR or sequencing. Importantly, PEA has greatly enhanced detection specificity over assays that rely on single antibody binding, such as flow cytometry or immunofluorescence (IF), due to its reliance on dual recognition by pairs of antibodies [30].

To date, this enhanced specificity has enabled multiplexed detection of antigens in 1 μL plasma samples [28] and even single-cell lysates [31]. Indeed, we recently demonstrated single-cell resolution for PEA-based protein measurements in multiwell plates while co-detecting RNA via qRT-PCR [31], echoing a previous report on a small panel of DNA, protein, and RNA targets [32], and in line with recent work that used PLA and qRT-PCR in reverse-emulsion droplets to examine the levels of a single protein and RNA [33]. In these examples, cellular RNA and protein expression were simultaneously profiled by splitting the lysate from a single cell (in half, three unequal portions (20:40:40), or half, respectively).

Although significant first steps, these demonstrations suffered from a few major shortcomings, most notably: (1) material loss associated with sample transfer, which reduces sensitivity and increases technical noise [31, 32]; and, (2) complicated workflows that are technically challenging to implement on multiple targets in a scalable, unified fashion, such as with an integrated fluidic circuit (IFC; like a C1 IFC [4, 21, 22]), reverse-emulsion droplets [7, 8], or microwells [34, 35]. As one potential alternative, Frei et al. recently developed a proximity ligation assay for RNA (PLAYR) to couple both RNA and protein quantification into a single mass cytometry readout [36]. While this enables rapid evaluation of RNA and protein across thousands of single cells, it is intrinsically limited by the number of heavy metal tags available.

To increase the number of probes and cells that can be simultaneously assayed, we have developed a new experimental method to detect and quantify several RNAs and proteins from the same single cell in one reaction chamber. Our approach utilizes reverse transcriptase as the DNA polymerase for both RT of cellular RNA and extension of PEA oligonucleotides to enable cDNA synthesis and PEA to proceed in a single series of reactions (see “Methods”). We implement our integrated profiling protocol on the C1 system to examine single cells from a human breast adenocarcinoma cell line (MCF7 cells) treated with phorbol-12-myristate-13-acetate (PMA), and benchmark our coupled RNA and protein measurements against in situ hybridizations and IF staining, respectively, as well as recombinant proteins, ERCC Spike-Ins, and population lysate dilutions (see “Methods”). Through a series of supervised and unsupervised computational analyses, we explore relationships between protein and RNA abundance. Overall, our method and coupled computational approaches provide a straightforward, scalable strategy for simultaneously studying the expression of many proteins and RNAs in single cells that can be adapted to a number of experimental configurations.

## Results and discussion

We sought to identify a means of integrating the PEA and cDNA synthesis workflows so that they could be performed in a single series of reactions. In examining both, we identified the possibility of coupling RT and PEA oligonucleotide extension into a single step by either reverse transcribing RNA with DNA polymerase or extending the hybridized DNA oligonucleotides in PEA with reverse transcriptase. Based on literature precedent [37], we devised a coupled PEA/specific (RNA) target amplification (STA) script for the C1 IFC that used the latter methodology. More specifically, our workflow is as follows (Fig. 1a): first, individual cells are isolated in the 96 capture sites of the C1 IFC. After washing, those cells are lysed with a buffer containing the PEA probes and incubated to achieve binding of the antibodies to their protein targets. Next, a DNA polymerization reaction is performed using reverse transcriptase to simultaneously extend the hybridized, complementary oligonucleotides conjugated to the PEA probes and reverse transcribe cellular RNA into cDNA using random primers. Importantly, we omit a DNAse I treatment for removing unwanted genomic DNA (gDNA) since it could destroy the single-stranded or double-stranded oligonucleotides on the PEA probes (when not hybridized or hybridized to a complementary probe, respectively). Instead, to reduce unwanted gDNA contamination, we designed our STA primers to span introns where possible (poly-dT priming could also be used), enabling RNA and gDNA to be differentiated via a melt-curve analysis of the qPCR product amplicons. After generating DNA reporters for protein and RNA abundance, multiplexed preamplification PCR is performed: for proteins, a universal primer pair amplifies all molecules generated by the oligonucleotide extension reaction; for STA, a mix of gene-specific primer pairs amplifies target cDNAs. Following harvest from the C1 IFC, the stable, amplified DNA libraries can be analyzed by high-throughput qPCR (or sequencing) to quantify both protein and RNA targets (Fig. 1b).

**Fig. 2:**
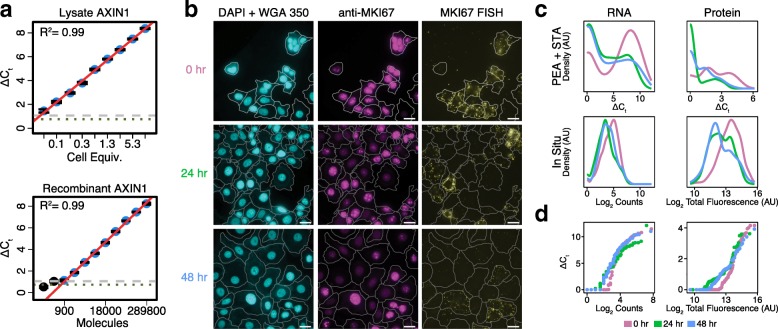
Benchmarking of a combined PEA/STA workflow: *AXIN1 and MKI67*. **a** Two-fold dilutions of bulk population lysate (*top*) and recombinant AXIN1 protein (*bottom*) were backloaded into the C1 IFC and detected using the same reactions conditions employed in the PEA/STA protocol. Each *data point* plotted is the average of eight replicates and *error bars* show the standard error of the mean. Points used for fitting the *red* trend line are colored *blue*. *Gray* (*green*) dashes show the level above which the probability for a detection event being real is *p* = 0.01 (0.05). **b**–**d** Validation of protein and RNA detection in single cells using a coupled PEA/STA script on the C1 throughout a PMA perturbation time course (0 hr = *purple*, 24 hr = *green*, 48 hr = *blue*). **b** RNA fluorescence in situ hybridization (RNA-FISH) and protein IF staining of MKI67 RNA and protein was performed to validate the C1-based, high-throughput RNA and protein measurements. *Cyan* (*left*) shows cell nuclei and boundaries, *magenta* MKI67 protein (*middle*), and *yellow* MKI67 RNA (*right*). Scale bars indicate 25 μm. **c** Qualitative agreement between the protein and RNA data obtained in situ and on the C1. Density distributions (each with their own arbitrary units) for MKI67 RNA (*left*) and protein (*right*) obtained via qPCR (*top*) or in situ (*bottom*) staining. **d** *Quantile-Quantile* (*Q-Q*) *plots* showing the range over which the PEA/STA measurements of MKI67 protein and RNA track linearly with IF staining or in situ hybridization

In order to evaluate the performance of our adapted PEA/STA reaction on the C1 IFC, we first examined dilutions of recombinant proteins and cell population lysates. The PEA probes, developed by Olink Proteomics, are intended for analysis of plasma samples and generally target secreted proteins. In previous work [31], we extended the list of PEA assays to include several intracellular targets. From this joint list, we selected 38 for our current study (Additional file 1: Table S1). To calibrate the sensitivity of the selected assays, we backloaded a dilution series containing recombinant protein targets for 25 of the 38 assays into the C1 IFC and processed it for PEA detection (see “Methods” and Additional file 1: Table S2). For most of those 25, such as a recombinant AXIN1 (Fig. 2a), we observed a wide linear dynamic range spanning an average 8 ± 2 two-fold dilutions (mean ± standard deviation; *n* = 23), suggesting effective PEA-based protein detection on the C1 (Additional files 1 and 2: Table S3 and Figure S1).

While this experiment enabled us to determine molecular sensitivity (Additional file 1: Table S4) and linearity for the majority of our assays, it did not provide information on whether they were quantitative about physiologically relevant, single-cell expression levels. To directly test this, we similarly backloaded population lysate dilutions into the C1 IFC and implemented our PEA/STA protocol. In analyzing our data, we found that 27 of the 38 PEA probes showed linear, above background responses in a range that included 1.3 cell equivalents of a bulk MCF7 cell lysate (Additional files 1 and 3: Table S5 and Figure S2a and “Methods”); we retained these and removed the others (Additional file 3: Figure S2b) for all subsequent analyses. Interestingly, we noted two failure modes (Additional file 3: Figure S2b): some PEA probes showed no signal while others appeared constantly saturated across all cell equivalents (but not in lysis buffer controls). For the former failure mode, we observed agreement between our population lysate and recombinant standard experiments (CSF3R_P and TP53_P; Additional files 2 and 3: Figure S1b and S2b). For the latter failure mode, one could envision decreasing probe concentration [38] or spiking in antibodies without DNA conjugates to achieve linearity, but both strategies would require further testing to determine their merits. For RNA, meanwhile, we only observed failure due to a lack of detection. Of the 96 RNAs we attempted to profile in parallel using gene-specific qPCR primer pairs (Additional file 1: Table S6 and S7), 89 showed linear responses to backloaded MCF7 lysate dilutions about the single-cell level (Additional files 1 and 4: Table S5 and Figure S3a); we retained these and removed the others (Additional file 4: Figure S3b) for all subsequent analyses. We propose that similar population lysate dilution assays should be used to determine the reliability of untested PEA or qPCR probes.

To directly test the performance of our combined single-cell PEA/STA quantification protocol on single cells, we chose to study MCF7 cells stimulated with PMA. Selecting this system allowed us to examine how RNA and protein levels, and their evolution over time, relate to important cellular behaviors [12, 31], as PMA has been shown to activate protein kinase C signaling, inhibit cell growth, and induce apoptosis in this human breast adenocarcinoma cell line [39]. Cells were exposed to PMA for 0 hr (untreated), 24 hr, or 48 hr. After, a single-cell suspension was loaded into a C1 IFC and processed according to the workflow depicted in Fig. 1 (see “Methods”). After culling cells that showed poor RNA expression (Additional file 1: Tables S8 and S9 and “Methods”), 87, 71, and 70 single cells remained for further analysis at the 0 hr, 24 hr, and 48 hr time points, respectively.

Before thoroughly analyzing our dataset, we first tested whether the patterns of heterogeneity we observed across multiple single cells using the C1 were biologically representative. For four genes (*MKI67*, *BIRC5*, *CASP8*, and *ICAM1*), we measured single-cell protein and RNA expression in situ using IF staining and RNA-FISH (see “Methods;” characteristic images shown in Fig. 2b, Additional files 5, 6, and 7: Figures S4a, S5a, and S6a, respectively). Figure 2c, Additional files 5, 6, and 7: Figures S4b, S5b, and S6b depict the RNA (left column) and protein (right column) distributions determined via PEA/STA (top row) or and in situ (bottom row) detection. In general, we observe good qualitative agreement with incongruences that can be attributed to the greater sensitivity of the in situ detection methods. Quantile-Quantile (Q-Q) plots (Fig. 2d, Additional files 5, 6, and 7: Figures S4c, S5c, and S6c for *MKI67*, *BIRC5*, *CASP8*, and *ICAM1*, respectively) show that our STA detection threshold approaches 4, 16, 8, and 4 RNA molecules for *MKI67*, *BIRC5*, *CASP8*, and *ICAM1*, respectively (assuming perfect RNA detection efficiency with RNA-FISH), with deviations likely due to inefficiencies in RT and subsequent PCR. We observed similar or greater sensitivity for STA using backloaded ERCC RNA Spike-Ins at known concentrations (see “Methods;” Additional file 1: Tables S4, S10, and S11, Additional file 8: Figure S7). Additionally, for BIRC5, CASP8, and ICAM1 RNA, the Q-Q plots show a vertical break between STA detected and undetected at or below the ΔC_t_ observed for 1.3 cell equivalents in the corresponding population lysate dilutions (Additional file 4: Figure S3a), possibly driven by our choice of normalization or the detection limits of our qPCR assays (see “Methods”). Interestingly, at high expression, we observe a plateauing of MKI67 STA detection but not RNA-FISH. Overall, STA has a larger dynamic range, potentially due to RT and/or PCR inefficiencies which can lead STA to overestimate the actual number of RNA molecules by which two cells differ. Meanwhile, in our protein measurements, we observe a substantially higher detection threshold for PEA and a slightly larger dynamic range for IF. The former observation may be due to PEA’s dual detection requirement, which limits the contribution of non-specific primary antibody binding that can skew in situ methods like IF. Taken together, these observations lead us to conclude that while in situ measurements are more sensitive than PEA/STA, the latter provides linear and highly multiplexable information on single-cell protein and RNA abundance.

We next examined the underlying structure of our dataset by performing a principal component analysis (PCA). PCA, using either the 27 proteins or 89 RNAs, distinguished PMA-treated from untreated cells, with protein providing clearer separation despite fewer targets evaluated (Additional file 9: Figure S8a, c, respectively). A random forest prediction algorithm (see “Methods”) supported this, yielding greater areas under the curve (AUC) for protein receiver operating characteristic (ROC) curves (0.98, 0.94, and 0.86 for protein versus 0.81, 0.80, and 0.57 for RNA at 0 hr, 24 hr, and 48 hr, respectively; Additional file 9: Figure S8b, d). Meanwhile, by using both protein and RNA data (Additional file 9: Figure S8e), we obtained AUCs of 0.99, 0.94, and 0.84 for the three time points, respectively (Additional file 9: Figure S8f). This suggests that, in certain instances, protein levels may be better biomarkers of environmental conditions [12], potentially due to either greater stability [16], a more direct role in cellular activity, or buffering from transcriptional noise [40] (also reflected in a lower average coefficient of variation (σ/μ); Additional file 10: Figure S9). This conclusion agrees with our previous results using split lysates in a different model system with a partially overlapping set of targets [31].

**Fig. 3:**
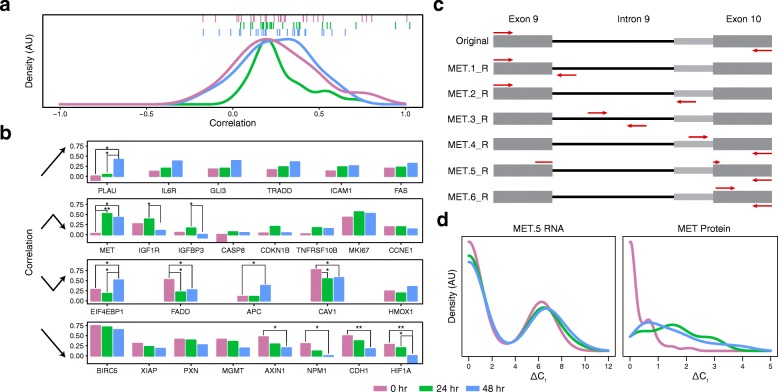
Time dynamics of the correlations between RNA and protein abundance. **a** The density of RNA:protein Spearman correlation coefficients (ρ) by time point, with *ticks* displaying individual genes from the three time points (0 hr = *purple*, 24 hr = *green*, 48 hr = *blue*). **b** Categorized by temporal correlation pattern, the correlations of the same gene across time points are juxtaposed. *, *p* value < 0.05; **, *p* value < 0.01. **c**, **d** Translational control of *MET* protein expression. **c** Approximate primer (*red*) locations for assays used to dissect splicing status of *MET* transcripts. RefSeq entries NM_000245.2 (short form) and NM_001127500.1 (long form) are the two reported splice isoforms of the *MET* transcript. The *thinner gray bar* indicates the segment included in the long form but not in the short form. Assay MET.1_R detects unspliced RNA; MET.2_R detects long form spliced RNA and, at reduced efficiency, unspliced RNA but was determined to not be quantitative by population lysate dilutions (Additional file 3: Figure S3b); MET.3_R detects unspliced RNA; MET.4_R detects long form spliced RNA and unspliced RNA at equal efficiency; MET.5_R detects short form spliced RNA and, at reduced efficiency, long form spliced RNA; MET.6_R detects both spliced forms and unspliced RNA with equal efficiency. **d** Distributions of spliced *MET* RNA (*left*) and *MET* protein at the three time points used in this study

To explore the interrelationship between RNA and protein expression, we next investigated correlations among the 27 genes targeted with both RNA and protein assays. In single cells, the correlation between RNA and protein quantities can be strongly influenced (and decoupled) by the transient nature of eukaryotic transcription [41], temporal delays between transcription and translation [3, 16, 19], differences in degradation rates [10, 15–17, 19], and technical noise [42]. For all RNA-protein pairs, we calculated Spearman correlation coefficients (ρ) at each time point (see “Methods”), obtaining an average (± standard deviation) correlation value of 0.25 (±0.23), 0.27 (±0.16), and 0.25 (±0.20) for the 0 hr, 24 hr, and 48 hr treatment time points, respectively (Fig. 3a). Intriguingly, the distribution of correlation values appears to tighten immediately after stimulation and then relax (*p* values from F test for variance are 0.08 and 0.30 for the 0 to 24 hr and 24 to 48 hr transitions, respectively). This trend may reflect the fact that, prior to stimulation, cellular activity across the targets assayed is more influenced by the aforementioned factors, which again come to dominate after a directed response to PMA.

When we investigated the relationship between each target’s mean expression, variance, and correlation (Additional file 10: Figure S9), we generally observed that RNAs with medium to high expression across cells had higher correlations prior to stimulation. After, the largest correlations appeared in RNAs with small to medium means and high cell-cell variance – this could reflect correlated activation of RNA and protein in only a subset of cells (bimodality), echoing previous findings in induced systems [3]. When focusing on significant changes in correlation (see “Methods”) between time points, we see that *CAV1* and *FADD* decrease in correlation within 24 hr, while the *MET* correlation increases. If we focus instead on the shift between 0 and 48 hr, we see that correlations between *AXIN1*, *CAV1*, *CDH1*, *FADD*, *HIF1A*, and *NPM1* RNA and protein are reduced, while those for *APC*, *EIF4EBP1*, *MET*, and *PLAU* increase. Finally, between 24 and 48 hr, *HIF1A*, *IGF1R*, and *IGFBP3* RNA and protein decrease in correlation while *EIF4EBP1* and *PLAU* increase (Fig. 3b). To better understand these PMA-induced shifts, we plotted the coefficients of variation for single-cell RNA and protein expression individually and found striking stability (Additional file 10: Figure S9) despite substantial variability between time points in the level of RNA expression among expressing cells and in the frequency of cells expressing a given protein (Additional files 11 and 12: Figures S10 and S11). Thus, even individual cellular perturbations can yield complex and heterogeneous RNA and protein responses across single cells (Fig. 3a, b, Additional files 10, 11, and 12: Figures S9, S10, and S11).

One particularly striking gene in Fig. 3b is *MET*, which has negligible correlation between protein and RNA levels in untreated cells (ρ = 0.03) but a strong positive correlation after PMA treatment (ρ = 0.53 and 0.42 for 24 and 48 hr cells, respectively). In re-investigating our STA data, we observed two distinct melting temperatures for the MET qPCR assay, indicating a complication due to the presence of splice variants. Because the libraries generated by preamplification are a stable archive, we re-analyzed them with new qPCR assays targeting additional sites contained within the original amplicons. For MET RNA, our preamplification primers were specific for exons 9 and 10, creating an amplicon that potentially spanned intron 9. Figure 3c shows this portion of the *MET* gene and the six assays we designed and deployed to interrogate the two isoforms previously known to exist in this segment of the MET transcript, as well as the unspliced transcript (primer sequences provided in Additional file 1: Table S7, all of which were determined to be quantitative from population dilution experiments except MET.2_R). Using a combination of the ΔC_t_ values and correlations between the various MET STA assays and MET_P (Additional file 13: Figure S12), we determined that the change in correlation between protein and RNA levels was primarily due to MET.5_R (short isoform, spliced) and MET.6_R (exon 10).

The distribution of different splice forms is evident in the scatterplot of MET.3_R (unspliced) versus MET.5_R (spliced) shown in Additional file 14: Figure S13. Across all three time points, a higher density of cells had only MET.3_R transcript (x-axis) than only MET.5_R transcript (y-axis), and an intermediate number of cells had both forms. Interestingly, the statistically significant increase in the proportion of cells with MET.3_R transcript at 24 hr (Fisher’s exact test *p* values = 0.0056 and 0.040 for comparing 24 h versus 0 and 48 hr, respectively) suggests that this transcript is actively being transcribed and processed during this time course. Still, because stop codons exist in the unspliced reading frame of intron 9, only the spliced forms of the MET transcript can be translated into MET protein (N.B. we assume that the MET PEA measurement, which relies on a polyclonal raised against the short MET isoform, primarily reflects the short isoform’s abundance, although further experiments will be needed to examine the sensitivity of the antibody for the long isoform and its contribution to the results).

Figure 3d shows the distributions of MET_P and MET.5_R (short isoform, spliced) for 0 hr, 24 hr, and 48 hr. For the protein, frequency of detection increased with PMA treatment (Benjamini–Hochberg (BH) adjusted Fisher’s exact *p* value = 1.1 × 10^−17^; Mann–Whitney U test for increased expression levels was not conducted since less than 10 unstimulated cells had expression above the limit of detection; Additional file 1: Table S12 provides differential expression for all targets between stimulated and unstimulated cells, while Additional file 1: Tables S13, S14, and S15 report targets differentially expressed between time points). Meanwhile there is no statistically significant change in the expression of spliced transcript (BH adjusted Fisher’s exact and Mann–Whitney U test *p* values = 0.90 and 0.088, respectively). A potential parsimonious explanation for this observation is that MET protein abundance is translationally regulated, which would account for the change in protein to RNA correlation from negligible to positive after PMA treatment. Intriguingly, putative control of MET protein levels by splicing (via skipping of exon 2) has previously been reported in many tissues [43]. This raises the question of whether the high proportion of single cells with only unspliced transcript observed in our study also reflects an aspect of *MET* regulation. While further experiments are needed to explore this, our observation of potential translational control emphasizes why, on these time scales and in this system, protein may be a better reporter of biological state than RNA.

Single-cell RNA expression profiling classically uses known protein biomarkers to pre-gate cells into subpopulations via FACS (and alternative methods) [1, 10, 21–23]. While this enables transcriptome-wide exploration of the differences between those discrete populations, each comparison represents a separate experiment. Here, because we quantified the levels of several RNAs and proteins in each single cell, we were able to gate our cells in silico on every measured RNA and protein to test if and how each marker bifurcated our data within a single experiment (Additional file 1: Table S16 and Additional file 15: Figure S14 a, b). Moreover, this allowed us to reverse-gate our data by RNA, enabling us to determine the impact of RNA expression on a host of expressed proteins. In examining the *MET* family, cells positive for the original MET_R STA assay (full length; Fig. 3c), not surprisingly, express MET.1_R (unspliced), MET.3_R (intron 9), and MET.4_R (long isoform and unspliced) at a higher frequency and MET.1_R, MET.3_R, MET.4_R, and MET.6_R (exon10) at higher levels. Additionally, dividing the data on MET_P detection shows that a MET_P expressing cell is more likely to have elevated expression of MET_R and MET.5_R (short isoform and spliced RNA), along with more frequent detection of MET.4_R and MET.6_R; reciprocally, MET.5_R expressing cells show elevated MET_P, MET.6_R, and MET_R. Here, the smaller *p* values associated with MET.5_R predicting MET_P suggests that, under certain conditions, RNA expression can be a better indicator of protein abundance than vice versa.

**Fig. 4:**
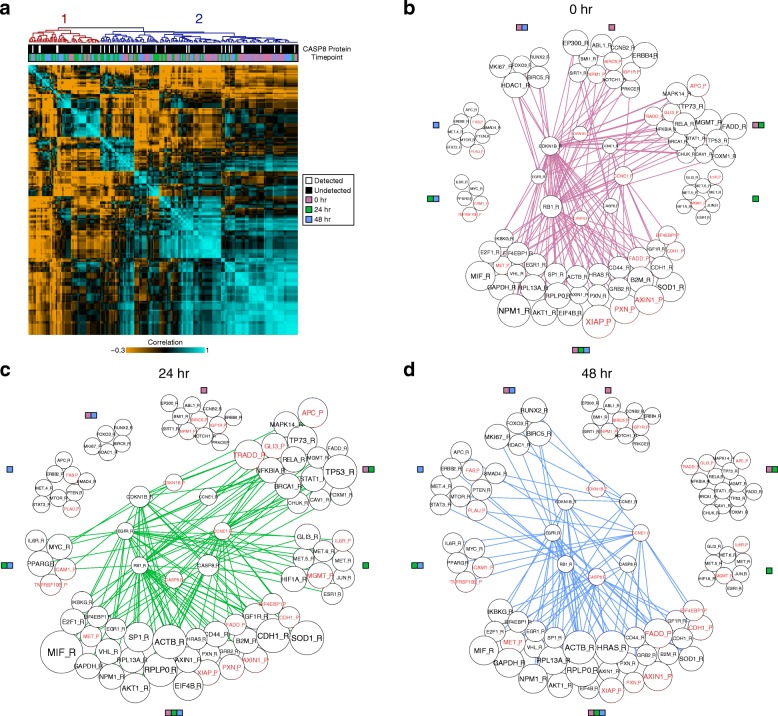
Determining intracellular circuits from known and in silico discovered networks. **a** *Heatmap* showing cell vs. cell correlation across a circuit scaled such that the maximum of both STA & PEA measurements are 1. The circuit is regulated by *CASP8*, with gates along the top indicating CASP8_P detection (*white*) or lack thereof (*black*) and time point (0 hr = *purple*, 24 hr = *green*, 48 hr = *blue*). The two major clusters are labeled 1 (*red*) and 2 (*blue*). **b**–**d** Changes in the Spearman correlation network from the known *CASP8* circuit measured at 0, 24, and 48 hr, nodes grouped by *edges. Edges* represent correlations greater than 0.3 between *CASP8* network and other targets. *Red text* indicates protein; *black text* indicates RNA; *number of edges* indicated by node size; *colored boxes* adjacent to the clusters indicate the time points for which a correlation coefficient greater than 0.3 exists between the target and the *CASP8* network

In addition to in silico gating, our data enabled directed questions of how the levels of upstream protein regulators and downstream RNA targets covary within known pathways. Of particular interest, given its role in apoptosis, is *CASP8*, a member of the caspase family. A survey of the literature revealed that *CCNE1*, *CDKN1B*, *EGFR*, and *RB1*, all profiled here, are downstream targets of *CASP8* [44–46]. A differential expression analysis after in silico gating on CASP8_P abundance showed a statistically significant decrease in the frequency of CDKN1B_P detection and elevated levels of RB1_R. When we examined the correlation structure of these downstream targets along with CASP8_R levels, we did not see statistically significant separation between cells in which CASP8_P is detected (white) and those in which it is not (black) (Fig. 4a, cluster membership 1 versus 2 denoted by red and blue labels, respectively, *p* value = 0.67, Fisher’s exact test). However, by overlaying time point metadata onto the clusters, we observed that cluster 2 is significantly enriched for unperturbed cells (*p* value = 0.00012, Fisher’s exact test). By growing a correlation network from this seed set of RNA and protein probes (see “Methods”), we were also able to observe stimulation-induced changes in the seed network’s members (e.g. edge degree = 0 at 0 hr but edge degree ≥ 1 over the 24 or 48 hr networks). This included cell cycle controllers (MYC_R, APC_R, PTEN_R, MTOR_R) and links to alternative modes of intracellular and intercellular regulation, such as cell surface (IL6R_R, IL6R_P, TNFRSF10B_P, ICAM1_P) and downstream signaling molecules (STAT3_R, SMAD4_R, PPARG_R) (Fig. 4b–d).

To better understand patterns in the genes correlated to the *CASP8* circuit, we conducted an unbiased functional analysis of enriched gene ontologies using the Database for Annotation, Visualization and Integrated Discovery (DAVID) [47] (see “Methods”). When analyzing genes that only correlate to the *CASP8* seed network in untreated cells, we observed an enrichment for annotations associated with cell division, cell cycle, and chromosome organization (BH adjusted *p* values < 10^−10^). Examining targets only correlated at 24 hr after PMA stimulation, we observe enrichments for DNA binding and transcription regulation (BH adjusted *p* values < 10^−10^), highlighting the cell state changes induced by PMA stimulation. Finally, when we examine genes only correlated to the *CASP8* circuit at 48 hr, we observe enrichments for cancer pathways (BH adjusted *p* values < 10^−7^), consistent with the breast adenocarcinoma origins of MCF7 cells.

To explore whether our quantification of RNA or protein abundance per single cell could be similarly used to inform the results of unsupervised protein or RNA analyses, we examined the extent to which observed RNA or protein level vectors correlated with the axes of variation in a protein or RNA PCA, respectively. Additional file 15: Figure S14c, d show correlations between the first two PCs over all protein or RNA targets and the expression of either ESR1_R or AXIN1_P, respectively [22]. Looking at Additional file 15: Figure S14c, we can see that ESR1_R levels correlate with separation in the protein-level PCA; considering the stimulation status of the cells (Additional file 9: Figure S8), this suggests that ESR1_R levels decrease with stimulation. A similar plot over RNA shows that AXIN1_P (Additional file 15: Figure S14d), meanwhile, correlates strongly with RNA PC1, independent of PMA, suggesting involvement in a stimulation-independent axis of variation. Although the clusters representative of stimulation condition are not well resolved in the RNA PCA, we envision that a similar analyses performed on PCAs showing greater separation will help guide hypothesis generation and follow-up experimentation in future studies [3, 4].

## Conclusions

We have presented a new method for simultaneously quantifying several proteins and RNAs from the same single cell in a single series of reactions, which we have validated with select in situ hybridization and IF experiments, as well as recombinant protein, bulk cell lysate, and ERCC Spike-In dilutions. Our integrated, single-chamber approach – which can be executed in an IFC – yields a highly multiplexed, coupled protein and RNA dataset that allows examination of the correlations and ties between several proteins and RNAs in mammalian cells. Here, we have used this workflow to study how these correlations and their expression underpinnings evolve over time in MCF7 cells under PMA perturbation. Moreover, since the unique dataset obtained via our generalized approach enabled many in silico experiments from a single in vitro experiment, we were able to discern how the levels of specific proteins and RNAs impact the expression of all other measured targets, saving time and money compared to conventional approaches [3–8, 10, 21–24].

Overall, our methodology yields cellular protein-level metadata that can be used to better interpret and annotate the results of unsupervised RNA analyses. Indeed, much of the excitement regarding single-cell genomic approaches, such as single-cell RNA-Seq [3–9, 21–24, 34], stems from their ability to help identify cell types, states, and circuits in a genome-wide manner. While putative biomarkers and drivers of these behaviors can be found by differential expression and gene set enrichment analyses, establishing the utility of these factors as biomarkers – e.g. if RNA X is differentially expressed between two subpopulations, will protein X also separate them? – requires follow-up labeling and/or perturbation experiments [3–8, 10, 21–24]. By conducting these experiments simultaneously, we have removed this roadblock. This could dramatically accelerate the discovery cycle, given complications associated with visualizing several RNAs in live cells [48], working with fixed cells [26], and the disconnect between RNA and protein levels [10, 15–17].

From an experimental perspective, current methods for sensitive detection of proteins in single cells require affinity reagents, such as the antibodies used here. Although our investigation analyzed 27 proteins, assaying a larger number per single cell is limited only by the availability and functionalization of high affinity antibodies. Further, the development of new or different protein-binding reagents (e.g. aptamers [29], nanobodies [49]), as well as the incorporation of established PEA-based methods for probing post-translational modifications and protein complexes [50], should further boost the power and promise of our approach. Ultimately, we envision that each of our analyses, performed using the method outlined here or variants that include immuno-PCR [51], single-cell RNA-Seq [3, 4, 10], or measurements of other cellular variables [10, 52, 53], will enable identification of biologically meaningful differences between cells and their molecular markers, generating unprecedented insights into the drivers of cellular heterogeneity.

## Methods

### Cell culture and drug treatment

Low-passage number human breast adenocarcinoma cell line MCF7 cells were maintained in high glucose Dulbecco’s Modified Eagle Medium supplemented with 10 % fetal bovine serum and incubated at 37 °C in a 5 % CO_2_ atmosphere. For PMA treatment, 3 mL of cell culture was seeded into each well of a 6-well plate at a density of 5 × 10^4^ cells/mL and the cells were allowed to settle. Subsequently, PMA was added to each of the wells at a final concentration of 1 μM for the treated cells and, after mixing, the multiwell plates were placed in the incubator for 24 hr or 48 hr. At time points 0 hr, 24 hr, and 48 hr post culture, cells were trypsinized, pelleted, and run on the C1 using a custom PEA/STA protocol.

RNA fluorescence in situ hybridization (RNA-FISH) and protein IF staining experiments were performed as previously described [3]. Briefly, 5 × 10^3^ cells were seeded into the interior wells of a black, imaging-grade glass-bottom 96-well plate and allowed to settle. Importantly, before adding cells, each well was cleaned with ethanol, treated with 100 μL of 0.01 % poly-L-lysine for 1 hr at 37 °C, washed, and dried overnight in a biosafety cabinet. After seeding cells, PMA was added to the wells at a final concentration of 1 μM for the treated 24 hr or 48 hr conditions and 0 μM for the 0 hr (untreated) condition. Prior to fixation, the culture media was replaced with 100 μL of Hanks’ Balanced Salt Solution supplemented with 1 mg/mL Wheat Germ Agglutinin 350 (WGA, Life Technologies, Thermo Fisher Scientific) for a 10 min incubation at 37 °C. The cells were then washed twice with phosphate buffered saline (PBS), fixed with 4 % formaldehyde in PBS at room temperature for 30 min, washed three times with PBS, and used for FISH and IF staining as described below.

### Selecting PEA/STA probes

PEA standard curves were generated (Additional file 3: Figure S2) using diluted MCF7 cell lysates ranging in average cellular content from 10.63 to 0.04 cells (full data table with ∆C_t_ measurements is provided as Additional file 1: Table S5 along with the corresponding STA data). While we evaluated a range of dilutions from 0.04 to 42.5 cell equivalents, we excluded the two highest dilutions (21.25 and 42.5 cell equivalents) because the PEA reaction displayed poor assay performance as evidenced by decreased amplification efficiency of the spike-in Extension Control and Oligo Reference probes depicted in Additional file 16: Figure S15. In Additional file 3: Figure S2, each red line represents the trend line generated from the points colored blue, with the y-axis depicting ∆C_t_ (as described further in “Data analysis: PEA/STA and calculating ∆C_t_”) relative to a lysis buffer background control (*n* = 8). The range used for the linear fit was found by evaluating every continuous range and picking the best R^2^ value with a cost of 0.03 for removing points, followed by manually extending or shortening the range where needed. Certain assays (e.g. EIF4EBP1_P) display a “hook” effect, which is evident when the concentration of target protein exceeds a threshold such that PEA probes occupy separate target molecules as opposed to the same one [38]. This reduction in the frequency of co-incidence binding events results in fewer DNA reporter molecules and thus a loss of signal. Probes in Additional file 3: Figure S2b were labeled unreliable and removed from later analysis due to either insensitivity, saturation, and/or failure to exceed the limit of detection within the physiological range (around 1.3 cell equivalents).

The results of this population lysate dilution experiment (see below) were corroborated with standard curves generated using 25 diluted recombinant proteins (Additional file 1: Table S3 and Additional file 2: Figure S1). Here, two probes (also filtered out by the above population lysate dilution experiments) did not display any signal (CSF3R_P and TP53_P, Additional file 2: Figure S1b) and thus were removed from all subsequent analyses.

In the same vein, a population lysate dilution experiment was designed to validate our STA probes (Additional file 1: Table S5 and Additional file 4: Figure S3). Probes that did not have a linear detection range or were not sensitive (Additional file 4: Figure S3b) were removed from later analysis.

### Recombinant protein and ERCC assay

Recombinant proteins (listed in Additional file 1: Table S2) were dissolved in a mixture of PBS and 1× C1 loading reagent. Serial dilutions of each protein were made using 1× C1 reagent in PBS. The only differences between this C1 run and the PEA/STA protocol for single cells was that the serially diluted proteins were backloaded into the C1 IFC using the outlet ports and cell wash buffer was loaded into the cell inlet instead of a single-cell suspension culture. ∆C_t_ for these samples (*n* = 8 for each dilution; Additional file 1: Table S3) was calculated in reference to wells with only lysis buffer (*n* = 8) and error bars are supplied plotted ± standard error of the mean (SEM).

Using the lysis buffer controls, we determined the mean and standard deviation of background for each target. These values enabled us to assign probabilities to detection. We defined our limit of detection as the fewest number of molecules which were detected at a confidence of greater than 0.01 in seven of the eight replicate measurements. Our limits of detection are presented as Additional file 1: Table S4 for recombinant proteins and ERCC Spike-Ins (described below). Detection is defined as a C_t_ value that has a probably less than 0.01 of being background noise.

ERCC Spike-Ins (ERCC RNA Spike-In Mix 1, Thermo Fisher Scientific 4456740) were also diluted in a mixture of PBS and 1× C1 loading reagent. Serial dilutions of the ERCCs were made using 1× C1 reagent in PBS. As with the recombinant proteins, the serially diluted ERCCs were backloaded into the C1 IFC using the outlet ports and cell wash buffer was loaded into the cell inlet instead of cell culture. ∆C_t_ for these samples (*n* = 8 for each dilution) was calculated in reference to wells with only lysis buffer (*n* = 8) or to a threshold C_t_ of 24 if undetected in lysis buffer alone, and error bars are supplied plotted ± standard error of the mean (SEM; Additional file 1: Table S11 and Additional file 8: Figure S7). Detection and limit of detection for each ERCC was also calculated as above for the recombinant proteins (Additional file 1: Table S4).

### Single-cell PEA/STA processing in C1 system

Cell processing and preparation for single-cell capture in the C1 were performed according to the manufacturer’s instructions (Fluidigm Corporation). The PEA/STA protocol for the analysis of single cells was implemented using the Script Builder™ feature of the C1 system. In particular, after capturing single cells in the C1 IFC, lysis of captured cells was performed in a lysis mix containing 1× lysis buffer (0.5 % NP-40, 50 mM Tris–HCl, pH 8.4, 1 mM EDTA), 8 % incubation solution (Olink Proteomics), 7.6 % incubation stabilizer (Olink Proteomics), 0.05 nM each PEA probe, and 1× C1 loading reagent (Fluidigm 100–5170). The lysis conditions were 37 °C for 120 min and 10 °C for 1 min. After lysis, a combined reverse transcriptase and PEA probe extension reaction was performed in a mix containing 1× RT master mix (Fluidigm 100–6299) and 1× C1 loading reagent using the conditions 42 °C for 60 min, 85 °C for 5 min, and 10 °C for 1 min. PCR was then performed in PCR mix containing 1× PreAmp Master Mix (Fluidigm Corporation, 100–5581), 50 nM of each preamplification primer, 0.1× PEA solution (Olink Proteomics), and 1× C1 loading reagent. The conditions for PCR were 95 °C for 5 min, 20 cycles of 96 °C for 20 s and 60 °C for 6 min, followed by 10 °C for 1 min. After harvesting from the C1, RNA expression was determined on the Biomark HD system using 2× Sso Fast EvaGreen Supermix with Low ROX (Bio-Rad 172–5212) and the script 96.96 Fast PCR + Melt.v2.pcl. The expression of proteins was determined with the Olink Proteomics assay setup and OLINK.pcl script on the Biomark HD system.

### RNA-FISH and protein IF staining

After fixation, RNA-FISH and IF were performed as previously described [3]. Briefly, the QuantiGene ViewRNA ISH Cell Assay (Affymetrix, Inc.) was performed with minor modifications. First, cells were not treated with Protease QS to keep the proteome intact for subsequent IF staining. Second, in order to stop the protocol, after hybridizing probes (*BIRC5* type 1, VA1-11137, *CASP8* type 1 VA1-12315-06, *ICAM1* type 1 VA1-12360-06, and *MKI67* type 1, VA1-11033, Affymetrix, Inc.), cells were washed 3× with FISH Wash Buffer (described in the QuantiGene ViewRNA ISH Cell Assay protocol) and stored in 6× Saline-Sodium Citrate buffer overnight at 4 °C. The following morning, cells were washed 2× with FISH Wash Buffer and the protocol was resumed. After hybridizing label probes, the cells were washed 3× with RNA-FISH Wash Buffer and 2× with PBS before incubating them for 1 hr at room temperature with a Block & Permeabilize Buffer (3 % IgG-Free Bovine Serum Albumin (BSA, Jackson ImmunoResearch), 0.2 % Triton-X 100 in PBS). The cells were then transferred to a primary staining solution of Block & Permeabilize Buffer supplemented with 4 μg/mL primary antibody (*BIRC5*: NB500-201, Novus Biologicals; *CASP8*: AF705, R&D Systems; *ICAM1*: AF720, R&D Systems; *MKI67*: ab15580, Abcam, Inc.) and incubated at 4 °C overnight. The following morning, cells were washed 3× in IF Wash Buffer (0.5 % BSA, 0.05 % Triton-X 100 in PBS) and developed in a secondary antibody staining solution containing Block & Permeabilize Buffer + 4 μg/mL secondary antibody (Alexa Fluor 488 goat anti-rabbit IgGH + L, A11034; Alexa Fluor 488 Donkey Anti-Sheep IgGH + L, A-11015; Alexa Fluor 488 Donkey Anti-Goat IgGH + L, A-11055, Thermo Fisher Scientific) at room temperature for 1 hr. Cells were then washed 2× in PBS and stained with DAPI (Affymetrix, Inc.; per the manufacturer’s recommendations) on a rocker for 1 min and imaged on an Olympus IX83 inverted microscope using the following excitation wavelengths: 405 nm – WGA and DAPI stains; 488 nm – secondary antibodies for IF; and 546 nm – type 1 FISH probes. Finally, to quantify RNA expression or total protein level, the images were processed using a custom Matlab script as previously reported [3]. The number of cells quantified at 0 hr, 24 hr, and 48 hr after treatment, respectively, for each experiment were: *BIRC5* – 1142, 1386, and 921 cells; *CASP8* – 5757, 3724, and 2066 cells; *ICAM1* – 5679, 2097, and 1548 cells; *MKI67* – 1699, 836, and 378 cells. Both raw density plots and Q-Q plots were generated to confirm qualitative agreement between in situ data generated by IF and RNA-FISH and the qPCR data generated by PEA/STA, respectively.

### Data analysis: PEA/STA and calculating ∆C_t_

The qPCR data for RNAs and proteins from the Biomark were analyzed by Fluidigm Real-time PCR analysis software using Linear (Derivative) Baseline Correction and Auto (Global) C_t_ Threshold Method. Exported C_t_ values (Additional file 1: Table S8) were then converted to ∆C_t_ values (Additional file 1: Table S9). For RNA, this was done using the equation of 24 minus C_t_ [2]. If the value was negative or if the qPCR never passed threshold, then the result was assigned 0 for undetected. Individual cells were characterized by the number of RNAs detected, with a median value of 54 RNAs detected per cell (57.5 after culling cells, 55 after culling cells and removing unreliable STA targets (Additional file 4: Figure S3b)). If less than 35 RNAs were expressed in a given cell after removing unreliable STA targets, then that cell was culled from the dataset. For protein, background was estimated from samples where no cell was captured in the C1, of which there were 5, 17, and 13 zero-cell samples at 0 hr, 24 hr, and 48 hr time points, respectively. Since there was no significant difference (by all time points pairwise T test) in the background C_t_ values when the time points were analyzed separately, the average value for all 35 zero-cell samples was used as the background value for each PEA probe, with protein C_t_ values above 24 (including undetected values of 999) set to a C_t_ of 24. Exported protein C_t_ values were then converted to ∆C_t_ values using each protein's average background value minus C_t_. If the resulting ∆C_t_ value was negative, it was assigned to 0.

### PCA and random forest classification

The culled data were used to conduct a PCA with the prcomp function in R, from which we observed separation based on time point. Ellipses were scaled to 68 % of the probability, or 1 standard deviation from the time point’s centroid. For every PCA, each target was first standardized to ensure equal representation.

For the random forest classification, we supplied the randomForest function from the randomForest package in R with all of the principal component scores for the “train” data, consisting of four-fifths of our samples randomly drawn with replacement. The model was then evaluated with the remaining one-fifth of the dataset to calculate sensitivities and specificities in a 1-vs.-Rest comparison, leveraging the prediction and performance functions from the ROCR package in R.

### Correlation analysis

Spearman correlation coefficients (ρ) were calculated for each of the genes that were evaluated as both RNA and protein. A Lilliefors test was conducted to confirm normality of the correlation distributions, after which differences in the time point distributions were evaluated using T and F tests, all of which returned negative for rejecting the null hypothesis of equal mean and variance, respectively. This, of course, is dependent on our sample size (27 genes in total), though we note a large deviation in variance from time point 0 to 24. Statistically significant changes in correlation were noted in the text and Fig. 3b if the delta correlation between any two time points had a probability less than 0.05 of being drawn from the null distribution. A null distribution was generated for each gene by mixing the time point labels for each cell 10,000 times and calculating a null correlation mean and standard deviation. These mean and standard deviation were used to calculate *p* values using the normal distribution. In Additional file 10: Figure S9, correlations are also shown as color values on plots of mean expression versus standard deviation. The dashed lines drawn on the plots indicate the standard deviation for a given mean if expression is only detected in 10 cells.

### Trajectory analysis

Cells were binned into four quadrants for every gene measured for both protein and RNA depending on the detection of both targets using a probability of 0.01 as a cutoff. Relative proportions of cells with low protein and RNA, low protein and high RNA, high protein and high RNA, and high protein and low RNA were clustered together for all genes with matched PEA/STA probes using a Spearman correlation. A distance metric of 0.75 was used to partition genes into similar clusters (Additional file 12: Figure S11, denoted by distinct colors). Representative plots from each cluster illustrate the changing fraction of cells within each of these gates across time.

### Differential expression and in silico gating

Prior to analyzing targets for differential expression, we examined our data to determine the most appropriate statistical test. Following precedent [4, 42], we attempted to fit our target expression distributions by perturbation time point to both a normal (two parameter) and a three-parameter model (normal + fraction expressing). From this analysis, 22/92, 25/93, and 20/90 were fit with a normal distribution (*p* value > 0.01) and 54/68, 44/57, and 31/51 were fit with the three-parameter model (*p* value > 0.01) for 0 hr, 24 hr, and 48 hr, respectively. Since only approximately two-thirds of the models passed a Chi-squared goodness-of-fit test, we decided to conduct two tests: (1) a Fisher’s exact test to determine if the proportion of cells expressing a target above the detection threshold was changing; and (2) a Mann–Whitney U test to determine if the distribution of expressing cells was changing significantly.

We then gated and bifurcated our data 116 times (the total number of quantitative targets measured by qPCR) based on detection of a given target and evaluated whether any of the remaining 115 targets were differentially represented in the two groups. Tests for difference in proportion (Fisher’s exact test) of cells expressing were conducted for every gate – target combination if the number of cells for which the target was undetected exceeded ten for the two populations. Complementarily, tests for difference in distribution (Mann–Whitney U test) among expressing cells were conducted for every gate – target combination if the number of cells for which the target was detected exceeded ten for the two populations. BH correction was then applied for each in silico experiment to adjust for false discoveries.

### Correlation network analysis

To determine the correlation network among our targets and observe how it changed following perturbation, we partitioned our data by time point and calculated Spearman correlation (ρ) between the seed *CASP8* network and every other target quantified. To determine a threshold for significant correlation, we generated a null distribution for each gene-gene pair by mixing the cell labels for each pair 10,000 times and calculating a null correlation mean and variance. From this analysis, the mean correlation for every gene-gene pair was less than 0.005 and the variance never exceeded 0.015. Based on those parameters, we calculated the threshold for 0.01 probability of being drawn from the background to be 0.29. Therefore, Spearman correlations over 0.3 were considered edges. We calculated edge-degree (the number of edges shared with the *CASP8* seed network) for each target for each network and sized the nodes according to this rank (Fig. 4). Lastly, we performed Gene Ontology enrichment using DAVID [47] across each set to assess the characteristics of the most strongly and sparsely regulated nodes and to test for the presence of expected connections.

## Additional files

Additional file 1:
**Supplementary Table.** Supplementary tables with legends in the first sheet called "Supplementary Table Legends". (XLSX 985 kb) Additional file 2: Figure S1. Standard protein probe curves using recombinant proteins. Two-fold dilutions of recombinant proteins were backloaded into the C1 IFC and processed according to the PEA/STA protocol. Shown here are the PEA measurements, with the *y-axis* values representing ∆C_t_ values from only lysis buffer. *Gray* (*green*) *dashes* show the level above which the probability for a detection event being real is *p* = 0.01 (0.05). Each *data point* plotted is the average of eight separate capture sites in the C1 IFC with *error bars* showing the standard error of the mean. Points used for fitting the *red* trend line are colored *blue*. Most probes evaluated with recombinants worked well (**a**) with the exception of CSF3R_P and TP53_P (**b**), whose lack of detection was also seen in the protein lysate dilutions (Additional file 3: Figure S2). (PDF 315 kb) Additional file 3: Figure S2. Standard protein probe curves using lysed and diluted MCF7 cells. Two-fold dilutions of population lysate were backloaded into the C1 IFC and processed according to the PEA/STA protocol. Shown here are the PEA measurements with the *y-axis* values representing ∆C_t_, which are calculated as the signal over a lysis buffer only control. Certain assays (e.g. EIF4EBP1_P) display a “hook” effect. The effect occurs when the concentration of a target protein exceeds a threshold beyond which PEA probes begin to occupy separate target molecules as opposed to the same one. This results in a reduction of signal due to a reduction in the number of proximal events. Each *data point* plotted is the average of eight separate capture sites in the C1 IFC with *error bars* showing the standard error of the mean. *Gray* (*green*) *dashes* show the level above which the probability for a detection event being real is *p* = 0.01 (0.05). Points used for fitting the *red* trend line are colored *blue*, the background plot color indicates which treatment cells were taken from (0 hr = *purple*, 24 hr = *green*, 48 hr = *blue*). Probes are categorized as (**a**) usable or (**b**) unusable. (PDF 557 kb) Additional file 4: Figure S3. Standard RNA probe curves using lysed and diluted MCF7 cells. Two-fold dilutions of population lysate were backloaded into the C1 IFC and processed according to the PEA/STA protocol. Shown here are the STA measurements with the *y-axis* values representing ∆C_t_ values from lysis buffer alone or a threshold value of 24 if undetected in pure lysate. Certain assays (e.g. ACTB_R) display a “hook” effect, as seen in Additional files 2 and 3: Figures S1 and S2, potentially due to reagent saturation. Each *data point* plotted is the average of eight separate capture sites in the C1 IFC with *error bars* showing the standard error of the mean. *Gray* (*green*) *dashes* show the level above which the probability for a detection event being real is *p* = 0.01 (0.05). Points used for fitting the *red* trend line are colored *blue*, the background plot color indicates which treatment cells were taken from (0 hr = *purple*, 24 hr = *green*, 48 hr = *blue*). Genes are categorized as (**a**) usable or (**b**) unusable. (PDF 1087 kb) Additional file 5: Figure S4. Additional benchmarking of a combined PEA/STA workflow: *BIRC5*. **a**–**c** Validation of protein and RNA detection in single cells using a coupled PEA/STA script on the C1 throughout a PMA perturbation time course (0 hr = *purple*, 24 hr = *green*, 48 hr = *blue*). **a** RNA-FISH and IF staining of *BIRC5* was performed to validate the C1-based, high-throughput RNA and protein measurements. *Cyan* (*left*) shows cell nuclei and boundaries, *magenta* BIRC5 protein (*middle*), and *yellow* BIRC5 RNA (*right*). Scale bars indicate 25 μm. **b** Qualitative agreement between the protein and RNA data obtained in situ and on the C1. Density distributions (each with their own arbitrary units) for BIRC5 RNA (*left*) and protein (*right*) obtained via qPCR (*top*) or in situ (*bottom*) staining. **c** *Q-Q plots* showing the range over which the PEA/STA measurements of *BIRC5* track linearly with IF staining or in situ hybridization for the same. (PDF 79799 kb) Additional file 6: Figure S5. Additional benchmarking of a combined PEA/STA workflow: *CASP8*. **a**–**c** Validation of protein and RNA detection in single cells using a coupled PEA/STA script on the C1 throughout a PMA perturbation time course (0 hr = *purple*, 24 hr = *green*, 48 hr = *blue*). **a** RNA-FISH and IF staining of *CASP8* was performed to validate the C1-based, high-throughput RNA and protein measurements. *Cyan* (*left*) shows cell nuclei and boundaries, *magenta* CASP8 protein (*middle*), and *yellow* CASP8 RNA (*right*). Scale bars indicate 25 μm. **b** Qualitative agreement between the protein and RNA data obtained in situ and on the C1. Density distributions (each with their own arbitrary units) for CASP8 RNA (*left*) and protein (*right*) obtained via qPCR (*top*) or in situ (*bottom*) staining. **c** *Q-Q plots* showing the range over which the PEA/STA measurements of *CASP8* track linearly with IF staining or in situ hybridization for the same. (PDF 124616 kb) Additional file 7: Figure S6. Additional benchmarking of a combined PEA/STA workflow: *ICAM1*. **a**–**c** Validation of protein and RNA detection in single cells using a coupled PEA/STA script on the C1 throughout a PMA perturbation time course (0 hr = *purple*, 24 hr = *green*, 48 hr = *blue*). **a** RNA-FISH and IF staining of *ICAM1* was performed to validate the C1-based, high-throughput RNA and protein measurements. *Cyan* (*left*) shows cell nuclei and boundaries, *magenta* ICAM1 protein (*middle*), and *yellow* ICAM1 RNA (*right*). Scale bars indicate 25 μm. **b** Qualitative agreement between the protein and RNA data obtained in situ and on the C1. Density distributions (each with their own arbitrary units) for ICAM1 RNA (*left*) and protein (*right*) obtained via qPCR (*top*) or in situ (*bottom*) staining. **c** *Q-Q plots* showing the range over which the PEA/STA measurements of *ICAM1* track linearly with IF staining or in situ hybridization for the same. (PDF 107675 kb) Additional file 8: Figure S7. Standard RNA probe curves using ERCC Spike-Ins. Two-fold dilutions of ERCC Spike-Ins were backloaded into the C1 IFC and processed according to the PEA/STA protocol. Shown here are the STA measurements with the *y-axis* values representing ∆C_t_ values from only lysis buffer or a threshold value of 24 if undetected in lysis buffer alone. *Plots* are ordered by decreasing concentration in the ERCC mix with bad fits arising around ERCC 14 (which corresponds to ~121 molecules loaded into the top dilution, Additional file 1: Table S10 and S11). Each *data point* plotted is the average of eight separate capture sites in the C1 IFC with *error bars* showing the standard error of the mean. *Gray* (*green*) *dashes* show the level above which the probability for a detection event being real is *p* = 0.01 (0.05). Points used for fitting the *red* trend line are colored *blue*. (PDF 1135 kb) Additional file 9: Figure S8. PCA separation of the various time points (0 hr = *purple*, 24 hr = *green*, 48 hr = *blue*). **a** A PCA over all quantitative protein targets and the corresponding ROC curves (**b**) for all three time points generated from random forest decision categorization with AUC of 0.98, 0.94, and 0.86 for 0 hr, 24 hr, and 48 hr, respectively. **c** A PCA over all quantitative RNA targets and the corresponding ROC curves (**d**) for all three time points generated from random forest decision categorization with AUC of 0.81, 0.80, and 0.57 for 0 hr, 24 hr, and 48 hr, respectively. **e** A PCA over all quantitative protein and RNA targets and the corresponding ROC curves (**f**) for all three time points generated from random forest decision categorization with AUC of 0.99, 0.94, and 0.84 for 0 hr, 24 hr, and 48 hr, respectively. For **a**, **c**, **e**, axis labels indicate which PC was used and what percent variance it explains. (PDF 269 kb) Additional file 10: Figure S9. Coefficient of variation colored by correlation. Genes quantified as both RNA and protein are plotted based on their standard deviation (*y-axis*) and mean (*x-axis*) on the *left* and *right* hand sides, respectively. The *plots* in the *top*, *middle*, and *bottom* rows are done at the 0 hr (*purple*), 24 hr (*green*), and 48 hr (*blue*) time points, respectively. *Dashed lines* follow the standard deviation of a gene that has only ten cells with uniform expression and the remaining cells have undetectable levels (strong bimodality). (PDF 233 kb) Additional file 11: Figure S10. Change in average, standard deviation, and frequency of expression. Density traces (each with their own arbitrary units) for change in mean, standard deviation, and frequency of expression are shown for genes quantified as both RNA (**a**) and protein (**b**). Each row depicts a time point transition (24 – 0 hr, 48 – 24 hr, 48 – 0 hr for the *top*, *middle*, and *bottom* rows, respectively) for every gene with at least two cells above detection in every time point for both RNA and protein (19 genes). The ticks display individual measurements from each time point transition. (PDF 508 kb) Additional file 12: Figure S11. Analyzing gene trajectories through a time course of PMA stimulation. Genes quantified as both RNA and protein were made binary for each value based on detection or lack thereof. The vector of the proportion of cells in the four *quadrants* of detected and undetected for RNA and protein across the three stimulation time points were clustered using Spearman correlation. Clusters are divided using a distance metric of 0.75; representative trajectories through time (depicted by color 0 hr = *purple*, 24 hr = *green*, 48 hr = *blue*) are shown for the genes evaluated in situ with the *bottom left quadrant* representing undetected protein and undetected RNA, the *bottom right quadrant* representing undetected protein and detected RNA, the *top left quadrant* representing detected protein and undetected RNA, and the *top right quadrant* representing detected protein and detected RNA. The size of the *dots* corresponds to the fraction of cells in that quadrant. (PDF 179 kb) Additional file 13: Figure S12. Correlation between protein and RNA: *MET*. Presented are the correlations of the various MET STAs with MET protein (by PEA) across time points (0 hr = *purple*, 24 hr = *green*, 48 hr = *blue*). We observe that the strong increase in correlation after stimulation is most noted in MET.5_R (spliced short isoform) and MET.6_R (exon 10), with the other STAs showing a modest increase in correlation at 24 hr that becomes negative by 48 hr. (PDF 122 kb) Additional file 14: Figure S13. Spliced vs. unspliced MET transcript. *Scatterplot* of MET.5_R (spliced, short isoform) vs. MET.3_R (unspliced) with *density curves* plotted along the axes. Time point indicated by color: 0 hr = *purple*, 24 hr = *green*, 48 hr = *blue*. (PDF 227 kb) Additional file 15: Figure S14. In silico gating of samples by both discrete and continuous methods. **a**, **b** *Heatmaps* show protein or RNA targets (*rows*) significantly dysregulated when applying protein or RNA expression gates (*columns*). Cooler colors indicate a decrease in expressing cells by a Fisher’s exact test (**a**) or the distribution of expressing cells by a Mann–Whitney U test (**b**) while warmer colors indicate the inverse. **c**, **d** In addition to discrete gating, unsupervised analyses, such as a PCA over protein (**c**) or RNA levels (**d**), can also be annotated by additional cellular observations (RNA or protein data, respectively). **c** ESR1_R expression shows a strong Spearman's ρ of 0.60 with PC2 on a PCA computed from the protein expression. **d** AXIN1_P expression shows a strong negative ρ of −0.68 with PC1 on a PCA computed over RNA expression. (PDF 964 kb) Additional file 16: Figure S15. Analyzing population lysate dilution data. The C_t_ values observed for the two PEA standards: the extension control (**a**) and oligo reference (**b**). Across time points (0 hr = *purple*, 24 hr = *green*, 48 hr = *blue*) and cell dilutions, the deviations from the lysis buffer control are quite small except for the 42.5 and 21.25 cell equivalent dilutions (0.05 and 0.01 probability cutoffs from the background shown in *green* and g*ray*, respectively). In these deviations are quite small except for the 42.5 and 21.25 cell equivalent dilutions. Therefore, those measurements are excluded from the dilution plots in Additional files 2, 3, and 4: Figures S1, S2, and S3. (PDF 143 kb)

## Abbreviations

AUC: Area under the curve
BH: Benjamini–Hochberg
BSA: Bovine serum albumin
cDNA: complementary DNA
DAVID: Database for annotation, visualization and integrated discovery
FACS: Fluorescence-activated cell sorting
gDNA: genomic DNA
IF: Immunofluorescence
IFC: Integrated fluidic circuit
PBS: Phosphate buffered saline
PCA: Principal component analysis
PEA: Proximity extension assay
PLA: Proximity ligation assay
PMA: Phorbol-12-myristate-13-acetate
Q-Q: quantile-quantile
qRT-PCR: Quantitative reverse transcription-polymerase chain reaction
RNA-FISH: RNA fluorescence in situ hybridization
ROC: Receiver operating characteristic
RT: Reverse transcription
STA: Specific (RNA) target amplification
WGA: Wheat Germ Agglutinin 350
