# Supplementary figures and images for: Multiplexed, targeted profiling of single-cell proteomes and transcriptomes in a single reaction
Source: Genome Biol. 2016 Sep 19;17:188. doi: 10.1186/s13059-016-1045-6 (PMC5027636; doi:10.1186/s13059-016-1045-6)

Figure S1

a

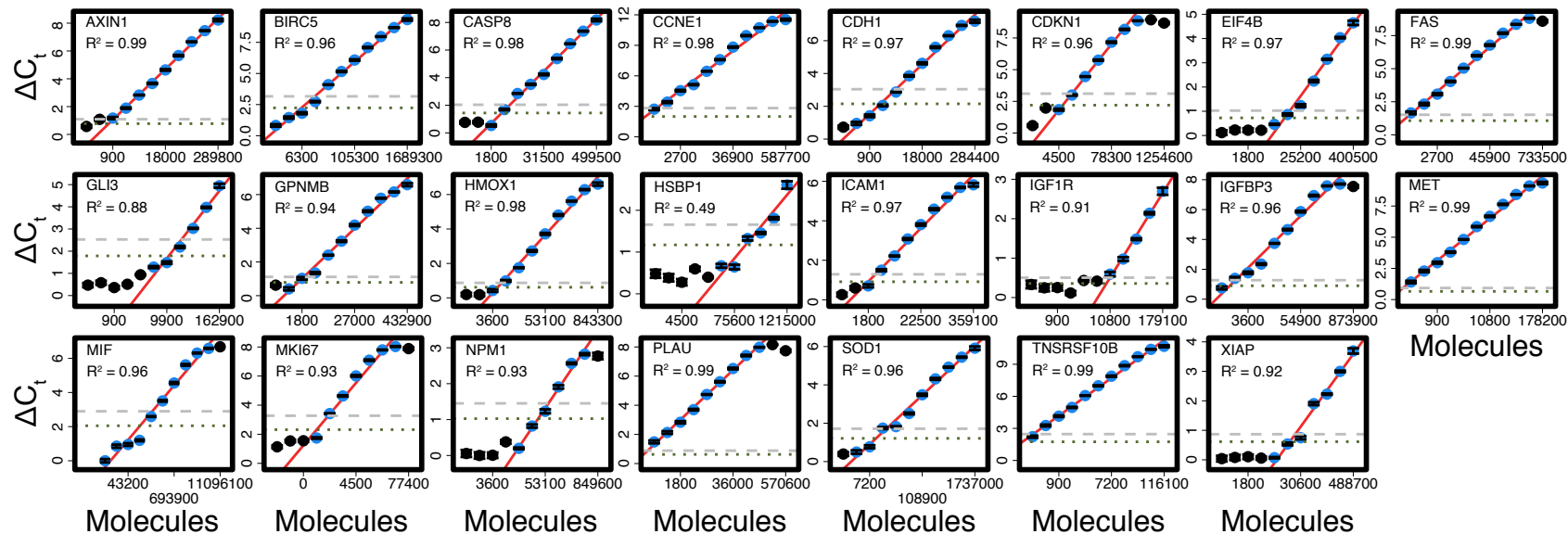

b

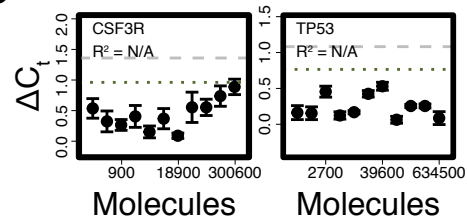

Supplement: Additional file 2: Figure S1. — Standard protein probe curves using recombinant proteins. Two-fold dilutions of recombinant proteins were backloaded into the C1 IFC and processed according to the PEA/STA protocol. Shown here are the PEA measurements, with the y-axis values representing ∆Ct values from only lysis buffer. Gray (green) dashes show the level above which the probability for a detection event being real is p = 0.01 (0.05). Each data point plotted is the average of eight separate capture sites in the C1 IFC with error bars showing the standard error of the mean. Points used for fitting the red trend line are colored blue. Most probes evaluated with recombinants worked well (a) with the exception of CSF3R_P and TP53_P (b), whose lack of detection was also seen in the protein lysate dilutions (Additional file 3: Figure S2). (PDF 315 kb) [file 13059_2016_1045_MOESM2_ESM.pdf]

Figure S2

a

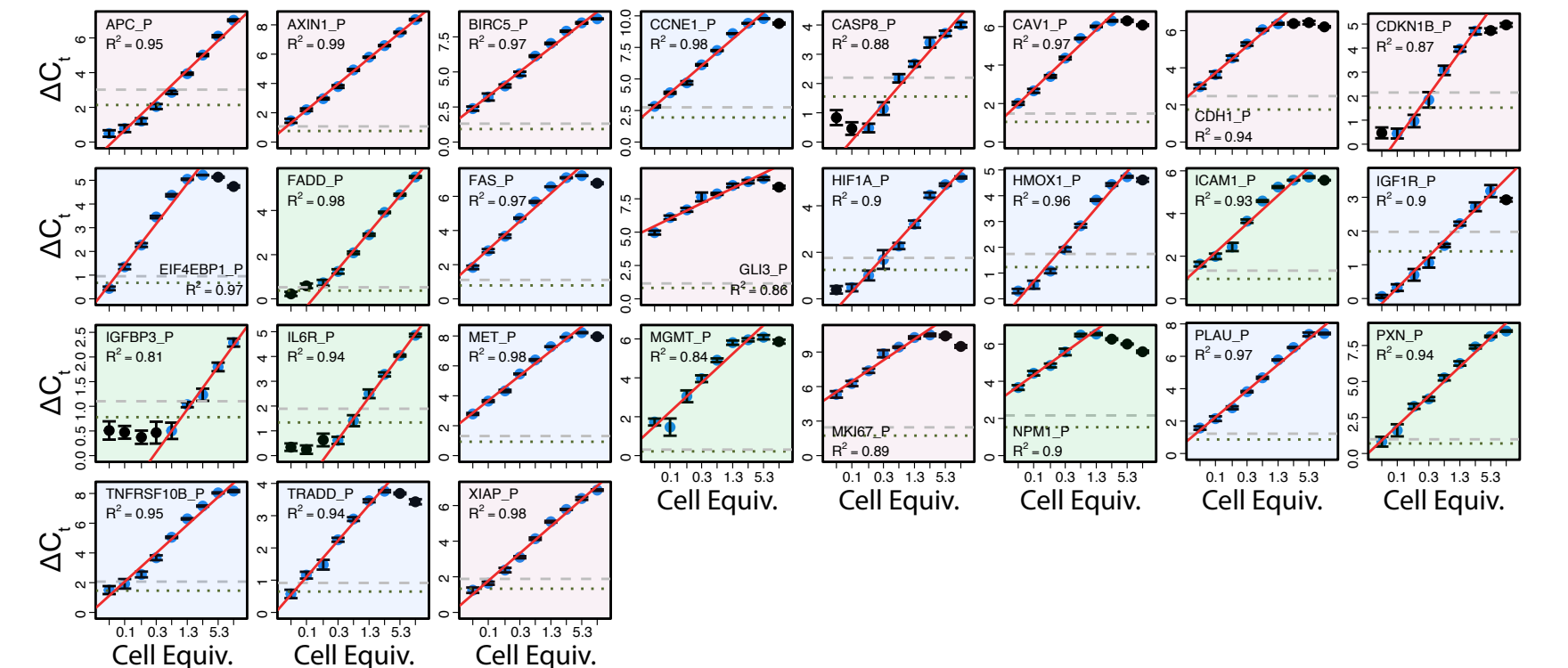

b

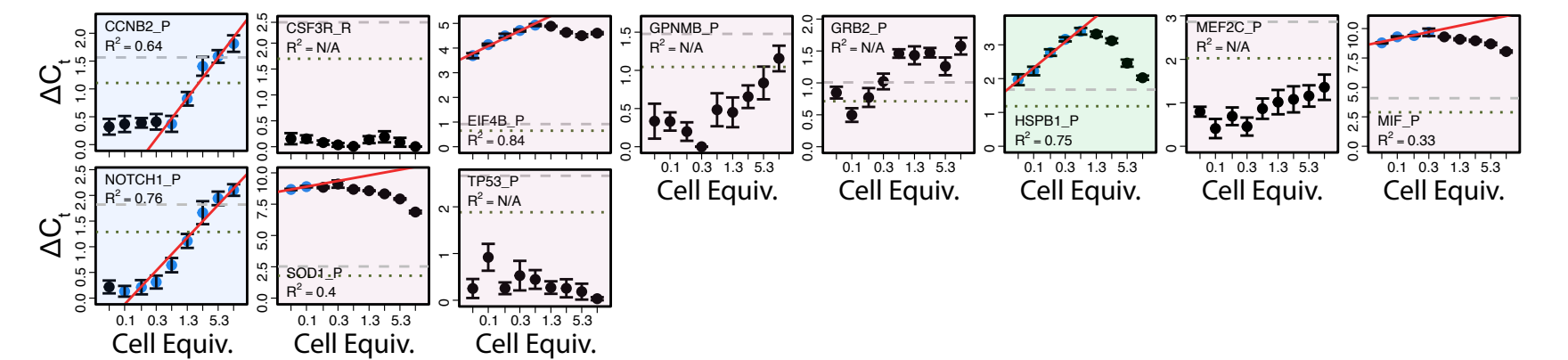

Supplement: Additional file 3: Figure S2. — Standard protein probe curves using lysed and diluted MCF7 cells. Two-fold dilutions of population lysate were backloaded into the C1 IFC and processed according to the PEA/STA protocol. Shown here are the PEA measurements with the y-axis values representing ∆Ct, which are calculated as the signal over a lysis buffer only control. Certain assays (e.g. EIF4EBP1_P) display a “hook” effect. The effect occurs when the concentration of a target protein exceeds a threshold beyond which PEA probes begin to occupy separate target molecules as opposed to the same one. This results in a reduction of signal due to a reduction in the number of proximal events. Each data point plotted is the average of eight separate capture sites in the C1 IFC with error bars showing the standard error of the mean. Gray (green) dashes show the level above which the probability for a detection event being real is p = 0.01 (0.05). Points used for fitting the red trend line are colored blue, the background plot color indicates which treatment cells were taken from (0 hr = purple, 24 hr = green, 48 hr = blue). Probes are categorized as (a) usable or (b) unusable. (PDF 557 kb) [file 13059_2016_1045_MOESM3_ESM.pdf]

Figure S3

a

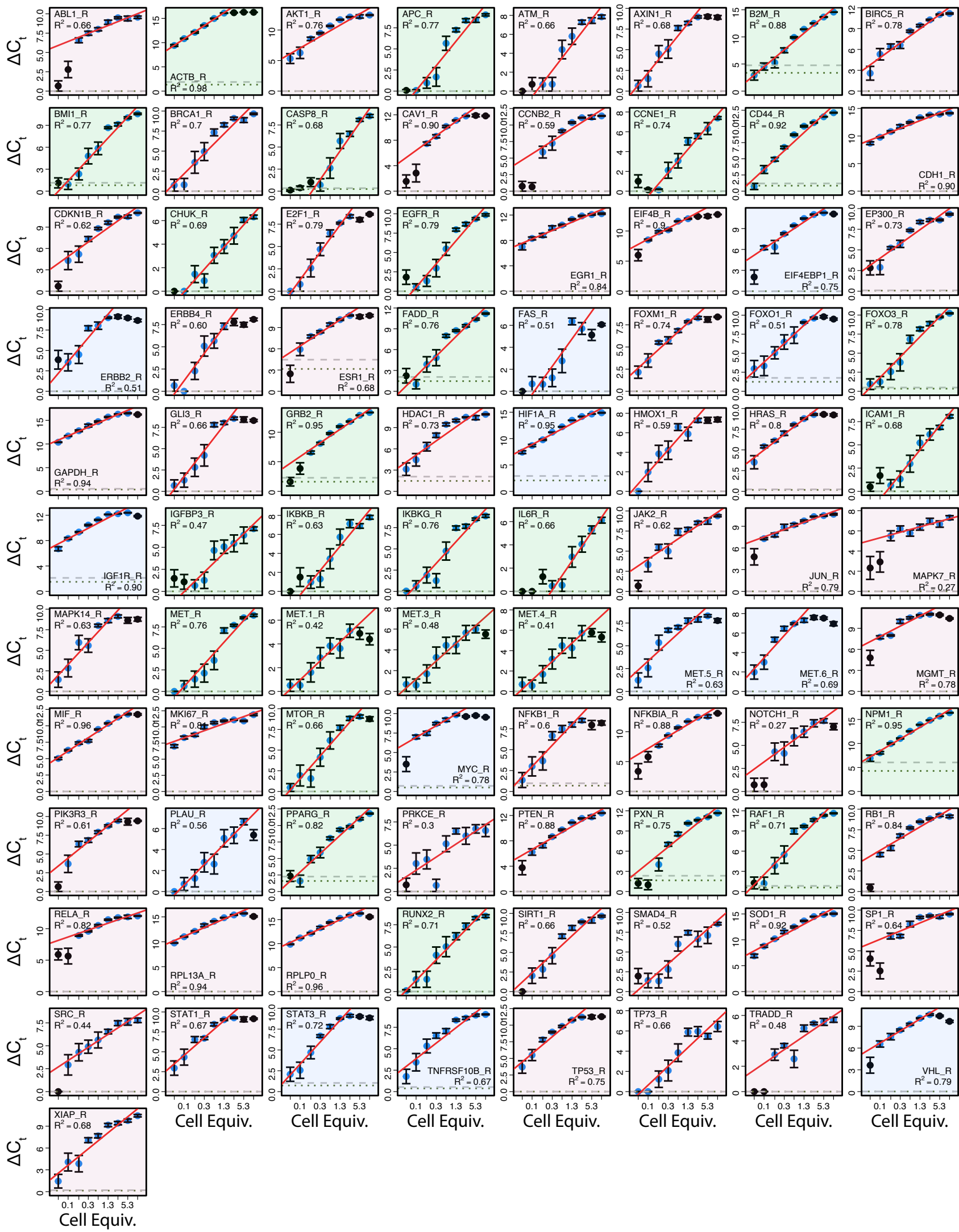

b

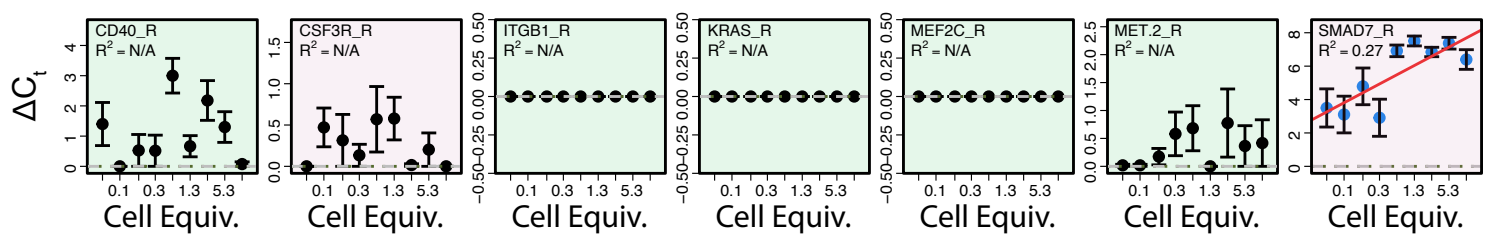

Supplement: Additional file 4: Figure S3. — Standard RNA probe curves using lysed and diluted MCF7 cells. Two-fold dilutions of population lysate were backloaded into the C1 IFC and processed according to the PEA/STA protocol. Shown here are the STA measurements with the y-axis values representing ∆Ct values from lysis buffer alone or a threshold value of 24 if undetected in pure lysate. Certain assays (e.g. ACTB_R) display a “hook” effect, as seen in Additional files 2 and 3: Figures S1 and S2, potentially due to reagent saturation. Each data point plotted is the average of eight separate capture sites in the C1 IFC with error bars showing the standard error of the mean. Gray (green) dashes show the level above which the probability for a detection event being real is p = 0.01 (0.05). Points used for fitting the red trend line are colored blue, the background plot color indicates which treatment cells were taken from (0 hr = purple, 24 hr = green, 48 hr = blue). Genes are categorized as (a) usable or (b) unusable. (PDF 1087 kb) [file 13059_2016_1045_MOESM4_ESM.pdf]

FIGURE S4

A

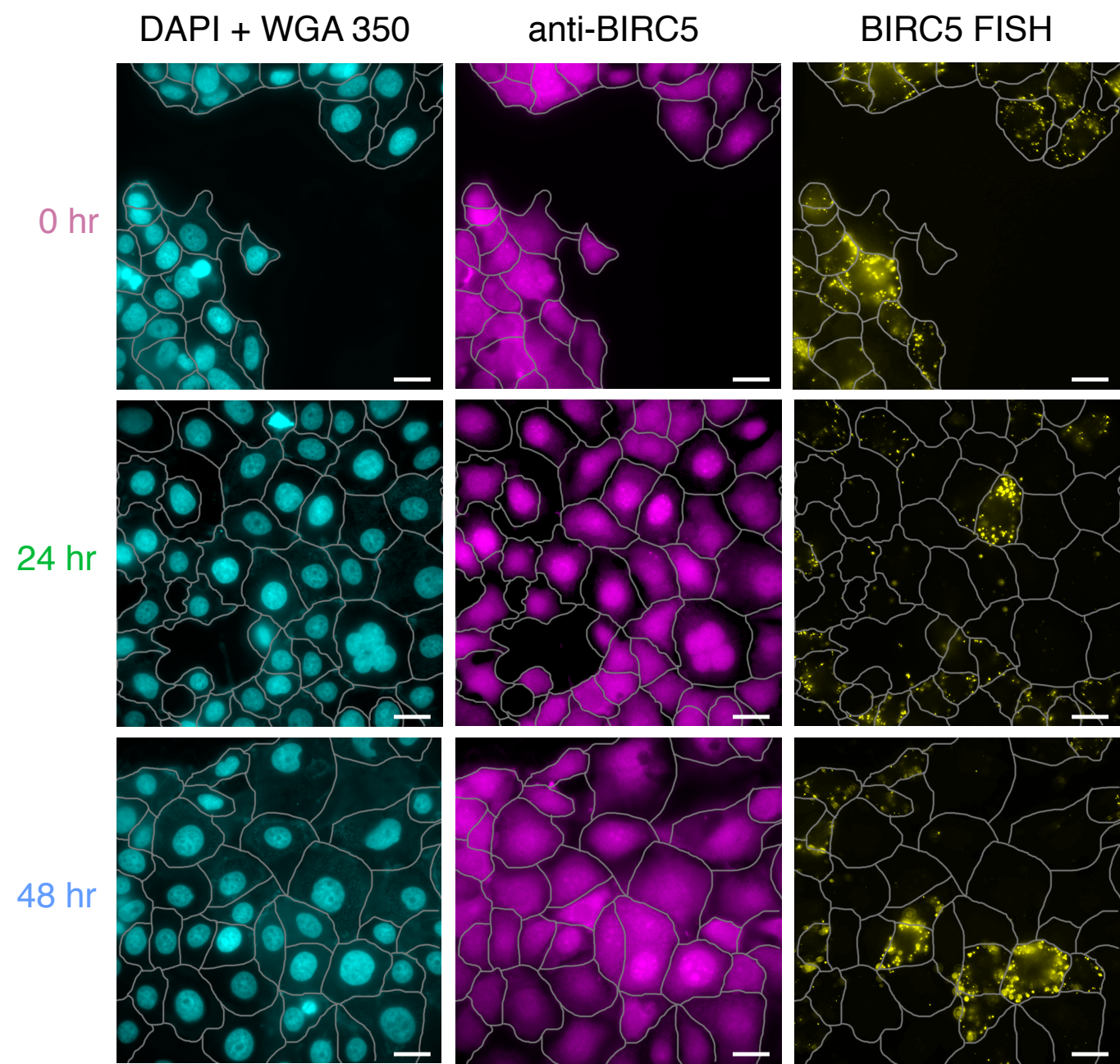

B

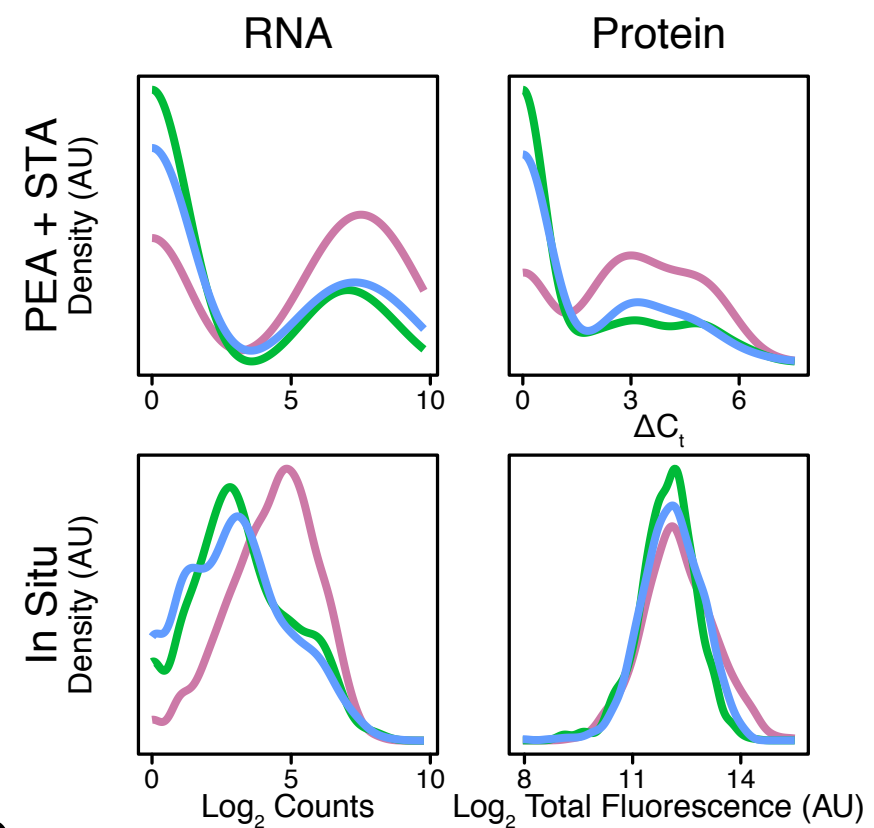

C

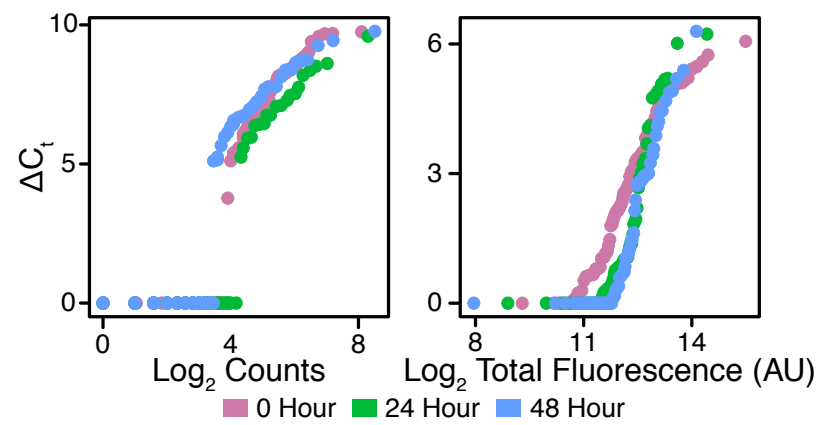

Supplement: Additional file 5: Figure S4. — Additional benchmarking of a combined PEA/STA workflow: BIRC5. a–c Validation of protein and RNA detection in single cells using a coupled PEA/STA script on the C1 throughout a PMA perturbation time course (0 hr = purple, 24 hr = green, 48 hr = blue). a RNA-FISH and IF staining of BIRC5 was performed to validate the C1-based, high-throughput RNA and protein measurements. Cyan (left) shows cell nuclei and boundaries, magenta BIRC5 protein (middle), and yellow BIRC5 RNA (right). Scale bars indicate 25 μm. b Qualitative agreement between the protein and RNA data obtained in situ and on the C1. Density distributions (each with their own arbitrary units) for BIRC5 RNA (left) and protein (right) obtained via qPCR (top) or in situ (bottom) staining. c Q-Q plots showing the range over which the PEA/STA measurements of BIRC5 track linearly with IF staining or in situ hybridization for the same. (PDF 79799 kb) [file 13059_2016_1045_MOESM5_ESM.pdf]

FIGURE S5

A

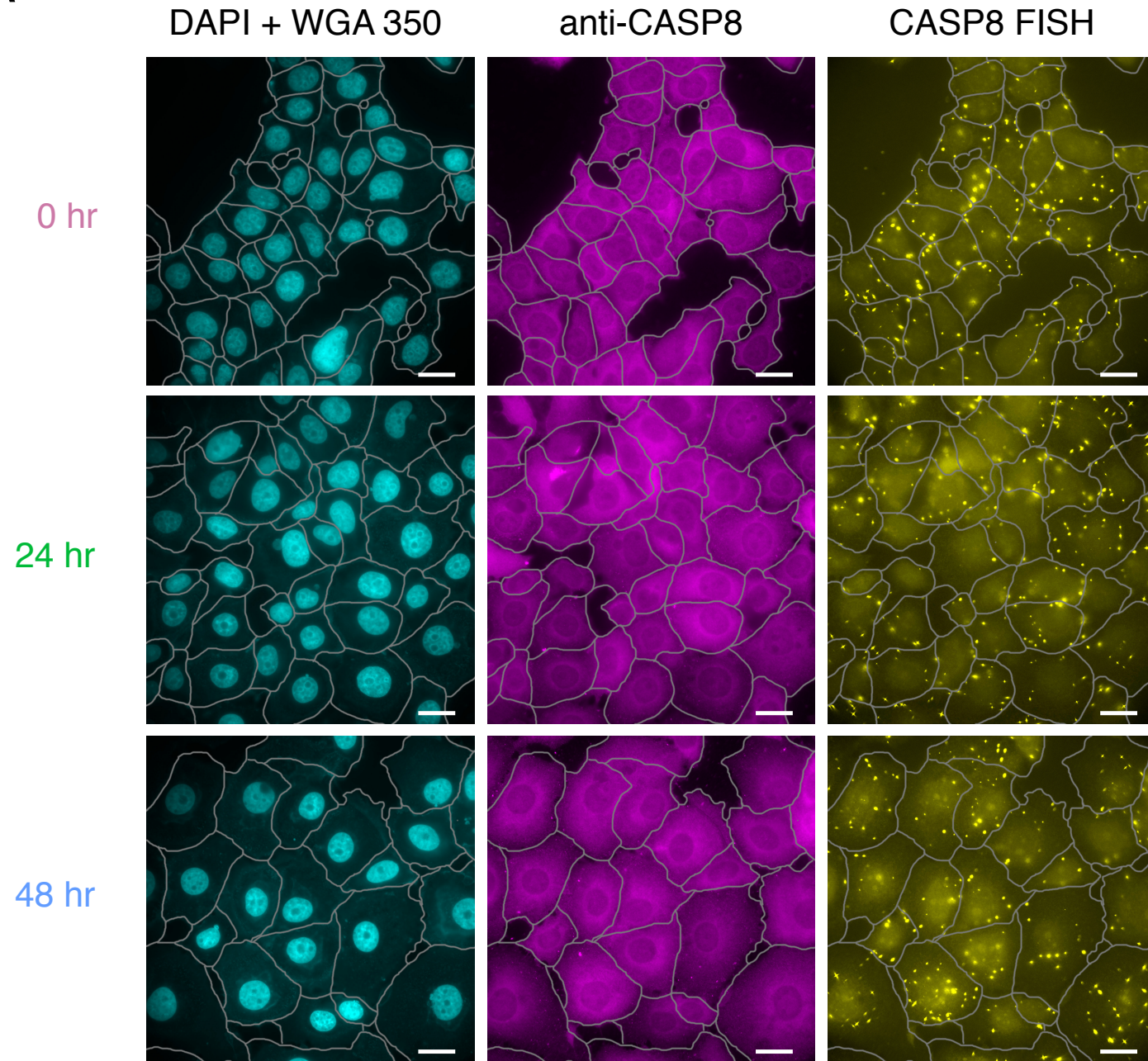

B

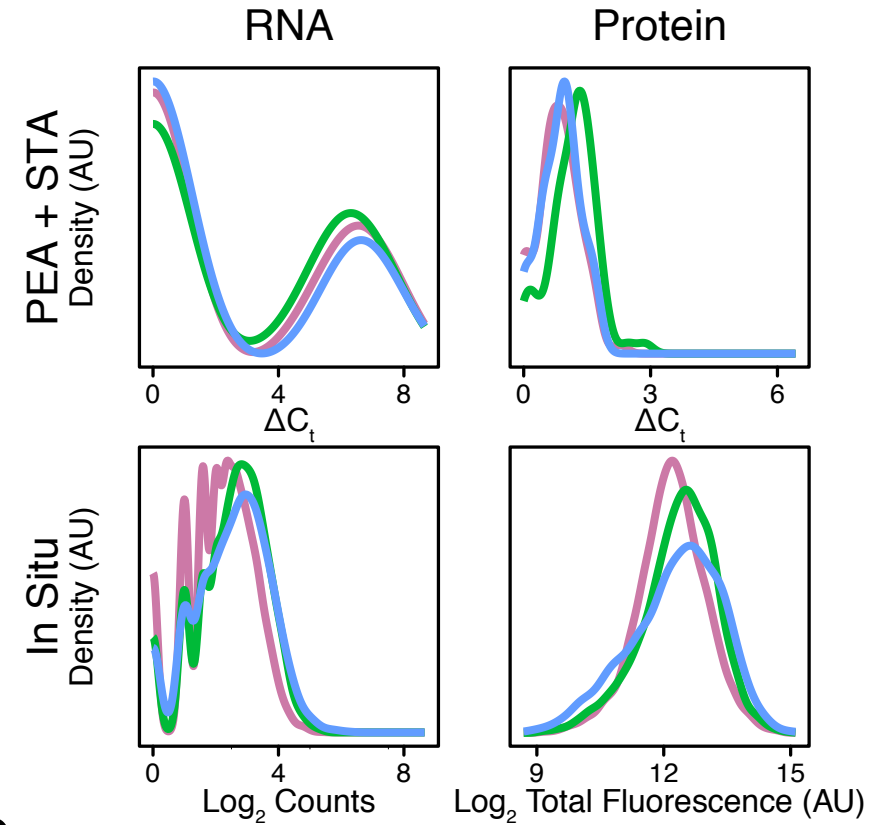

C

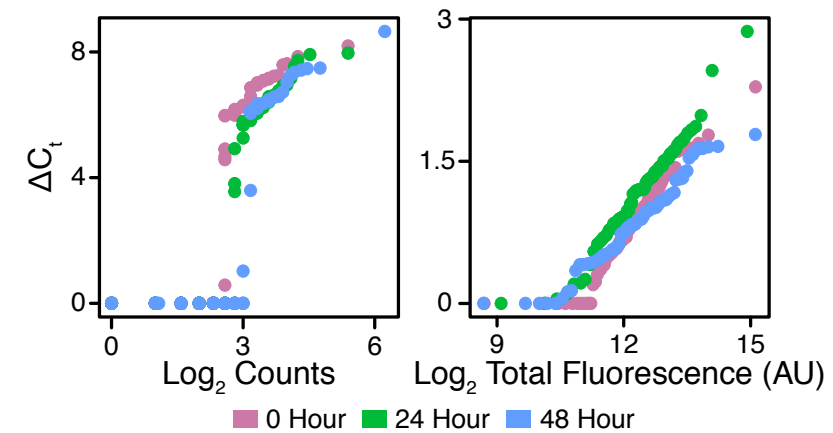

Supplement: Additional file 6: Figure S5. — Additional benchmarking of a combined PEA/STA workflow: CASP8. a–c Validation of protein and RNA detection in single cells using a coupled PEA/STA script on the C1 throughout a PMA perturbation time course (0 hr = purple, 24 hr = green, 48 hr = blue). a RNA-FISH and IF staining of CASP8 was performed to validate the C1-based, high-throughput RNA and protein measurements. Cyan (left) shows cell nuclei and boundaries, magenta CASP8 protein (middle), and yellow CASP8 RNA (right). Scale bars indicate 25 μm. b Qualitative agreement between the protein and RNA data obtained in situ and on the C1. Density distributions (each with their own arbitrary units) for CASP8 RNA (left) and protein (right) obtained via qPCR (top) or in situ (bottom) staining. c Q-Q plots showing the range over which the PEA/STA measurements of CASP8 track linearly with IF staining or in situ hybridization for the same. (PDF 124616 kb) [file 13059_2016_1045_MOESM6_ESM.pdf]

FIGURE S6

A

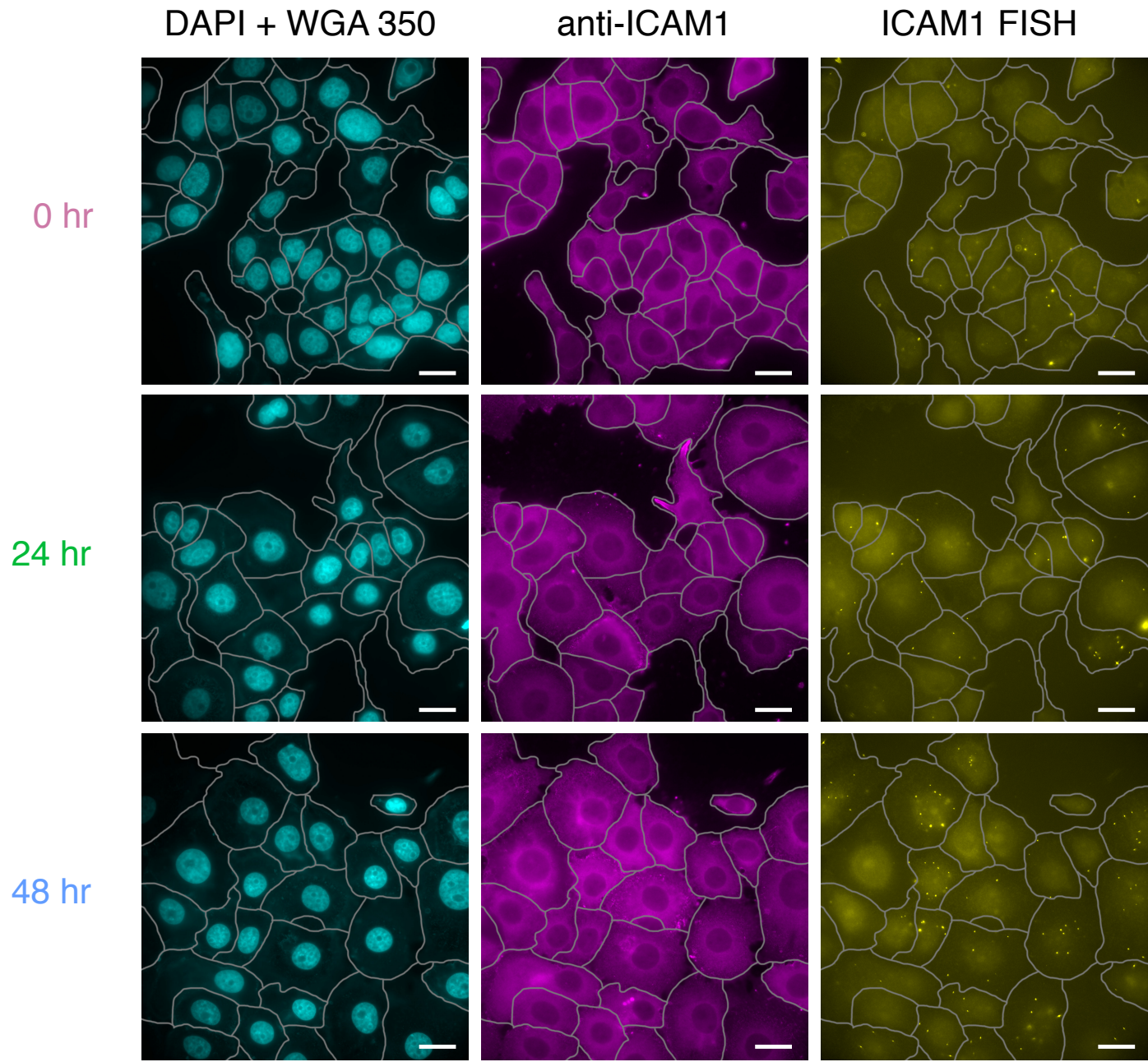

B

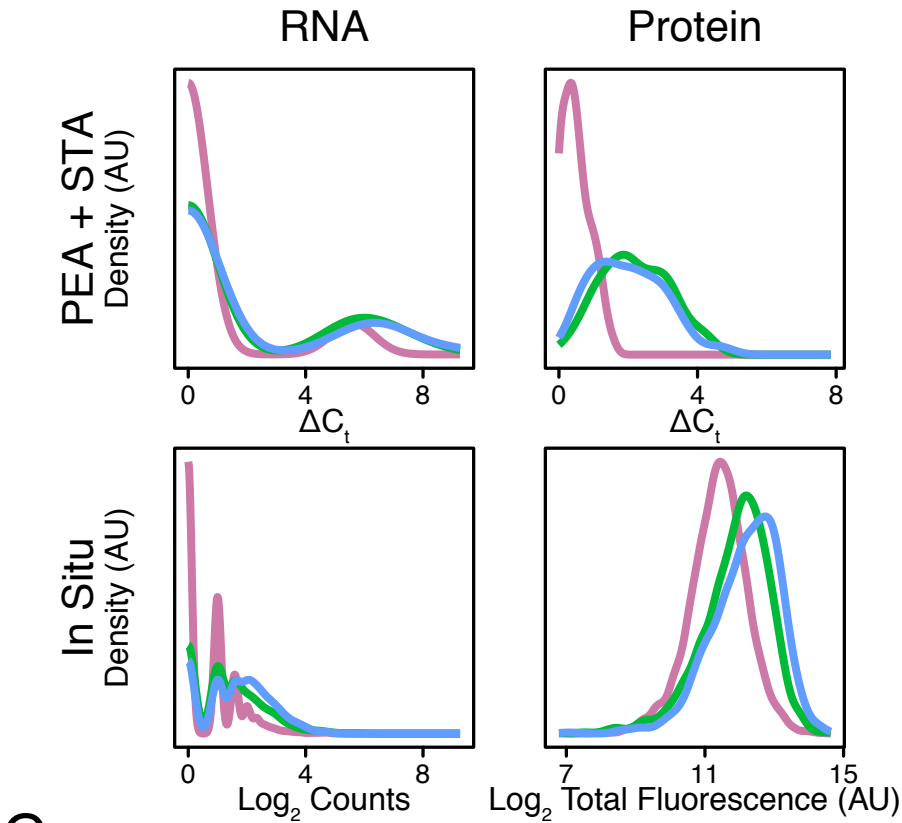

C

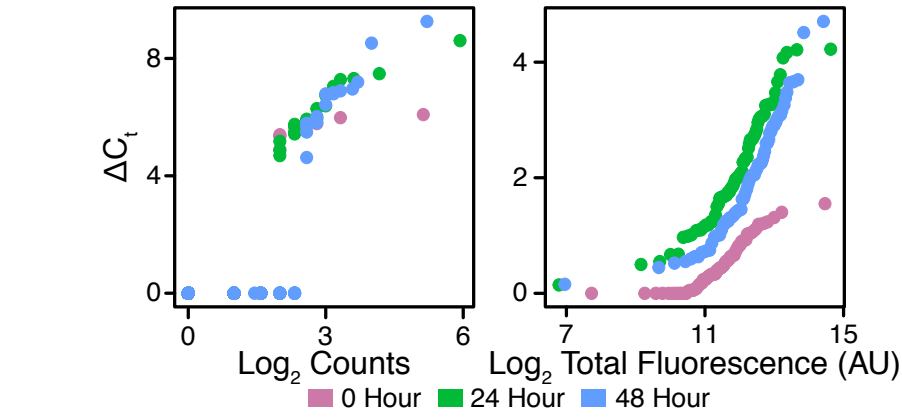

Supplement: Additional file 7: Figure S6. — Additional benchmarking of a combined PEA/STA workflow: ICAM1. a–c Validation of protein and RNA detection in single cells using a coupled PEA/STA script on the C1 throughout a PMA perturbation time course (0 hr = purple, 24 hr = green, 48 hr = blue). a RNA-FISH and IF staining of ICAM1 was performed to validate the C1-based, high-throughput RNA and protein measurements. Cyan (left) shows cell nuclei and boundaries, magenta ICAM1 protein (middle), and yellow ICAM1 RNA (right). Scale bars indicate 25 μm. b Qualitative agreement between the protein and RNA data obtained in situ and on the C1. Density distributions (each with their own arbitrary units) for ICAM1 RNA (left) and protein (right) obtained via qPCR (top) or in situ (bottom) staining. c Q-Q plots showing the range over which the PEA/STA measurements of ICAM1 track linearly with IF staining or in situ hybridization for the same. (PDF 107675 kb) [file 13059_2016_1045_MOESM7_ESM.pdf]

Figure S7

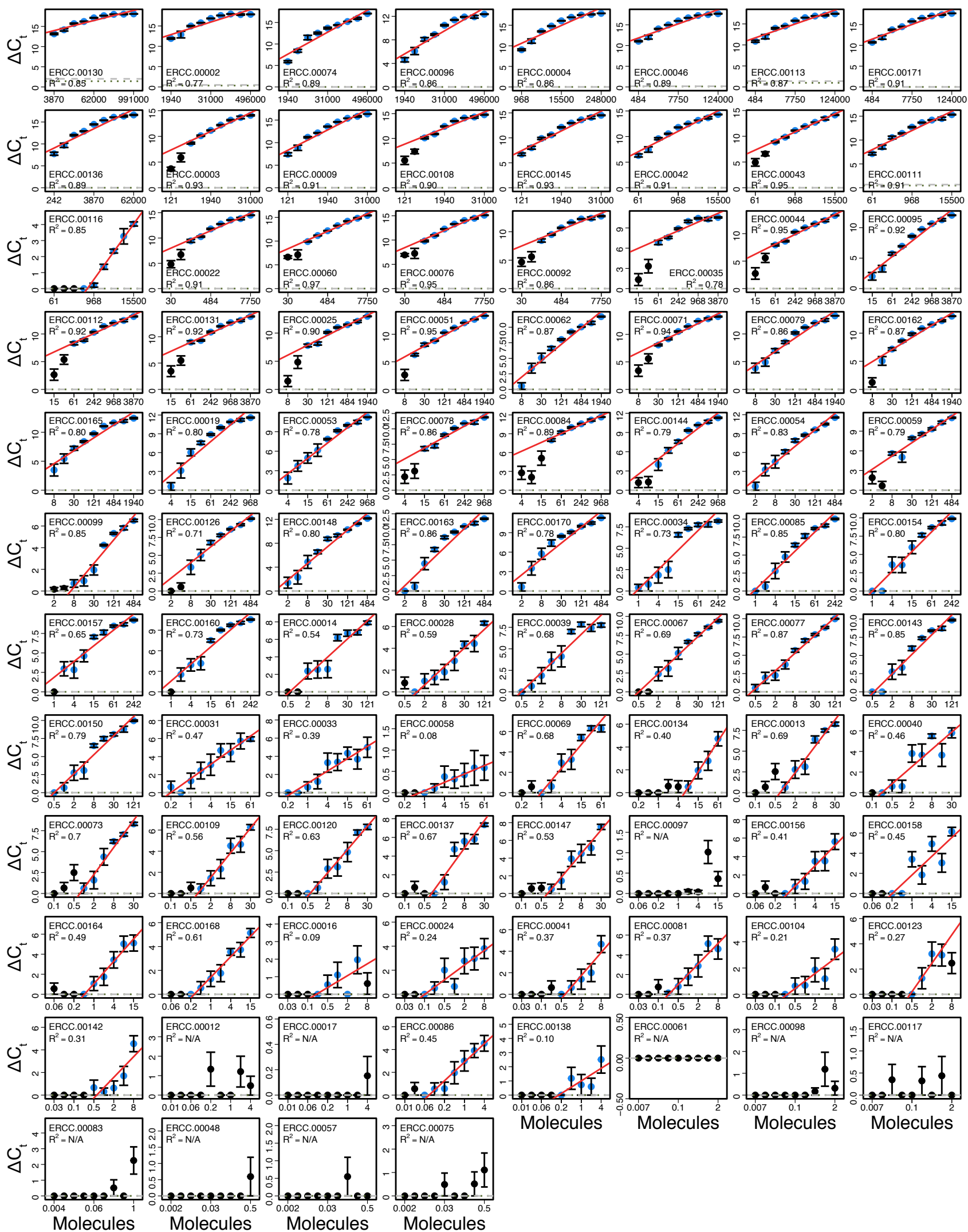

Supplement: Additional file 8: Figure S7. — Standard RNA probe curves using ERCC Spike-Ins. Two-fold dilutions of ERCC Spike-Ins were backloaded into the C1 IFC and processed according to the PEA/STA protocol. Shown here are the STA measurements with the y-axis values representing ∆Ct values from only lysis buffer or a threshold value of 24 if undetected in lysis buffer alone. Plots are ordered by decreasing concentration in the ERCC mix with bad fits arising around ERCC 14 (which corresponds to ~121 molecules loaded into the top dilution, Additional file 1: Table S10 and S11). Each data point plotted is the average of eight separate capture sites in the C1 IFC with error bars showing the standard error of the mean. Gray (green) dashes show the level above which the probability for a detection event being real is p = 0.01 (0.05). Points used for fitting the red trend line are colored blue. (PDF 1135 kb) [file 13059_2016_1045_MOESM8_ESM.pdf]

FIGURE S8

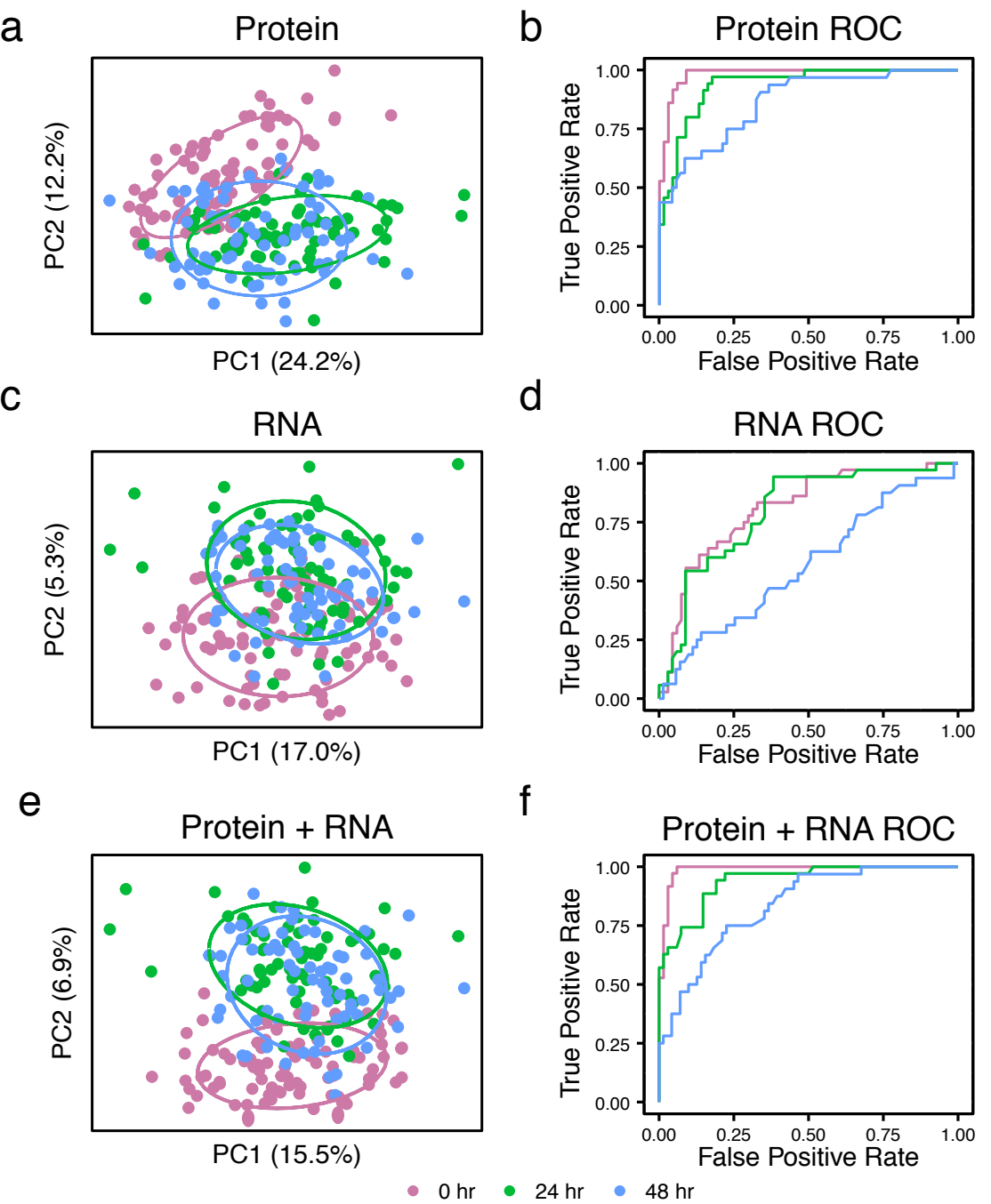

Supplement: Additional file 9: Figure S8. — PCA separation of the various time points (0 hr = purple, 24 hr = green, 48 hr = blue). a A PCA over all quantitative protein targets and the corresponding ROC curves (b) for all three time points generated from random forest decision categorization with AUC of 0.98, 0.94, and 0.86 for 0 hr, 24 hr, and 48 hr, respectively. c A PCA over all quantitative RNA targets and the corresponding ROC curves (d) for all three time points generated from random forest decision categorization with AUC of 0.81, 0.80, and 0.57 for 0 hr, 24 hr, and 48 hr, respectively. e A PCA over all quantitative protein and RNA targets and the corresponding ROC curves (f) for all three time points generated from random forest decision categorization with AUC of 0.99, 0.94, and 0.84 for 0 hr, 24 hr, and 48 hr, respectively. For a, c, e, axis labels indicate which PC was used and what percent variance it explains. (PDF 269 kb) [file 13059_2016_1045_MOESM9_ESM.pdf]

FIGURE S9

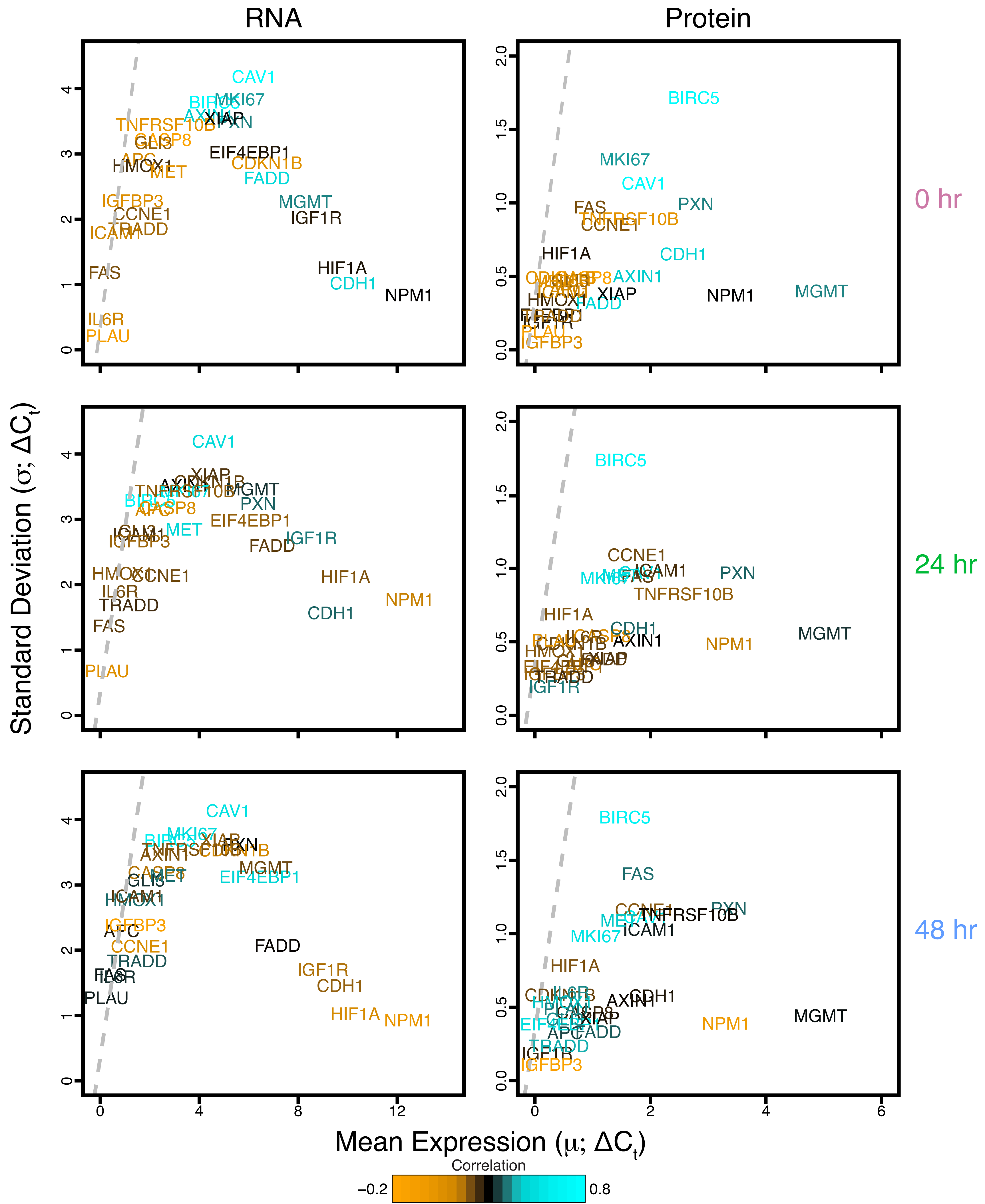

Supplement: Additional file 10: Figure S9. — Coefficient of variation colored by correlation. Genes quantified as both RNA and protein are plotted based on their standard deviation (y-axis) and mean (x-axis) on the left and right hand sides, respectively. The plots in the top, middle, and bottom rows are done at the 0 hr (purple), 24 hr (green), and 48 hr (blue) time points, respectively. Dashed lines follow the standard deviation of a gene that has only ten cells with uniform expression and the remaining cells have undetectable levels (strong bimodality). (PDF 233 kb) [file 13059_2016_1045_MOESM10_ESM.pdf]

FIGURE S10

a

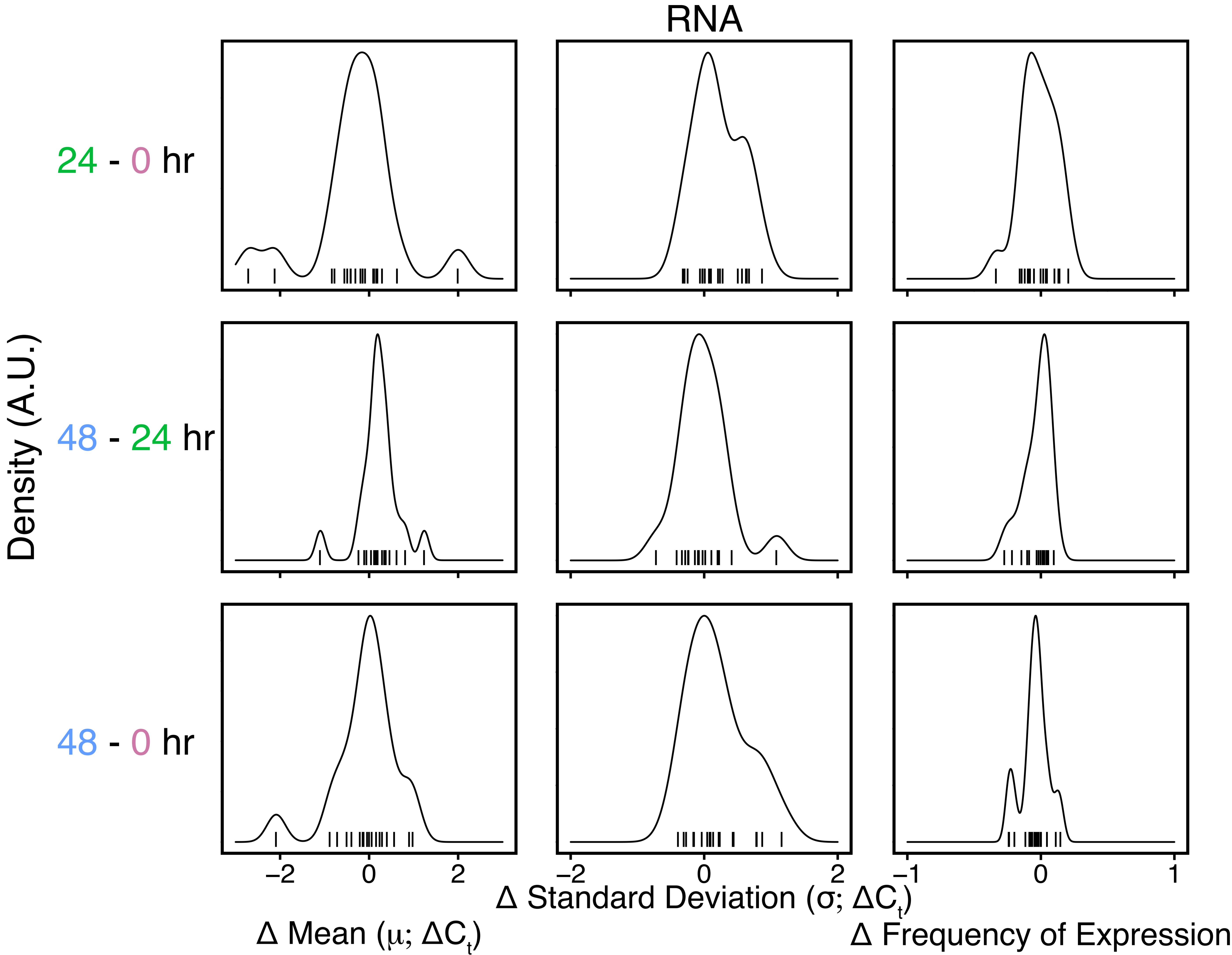

b

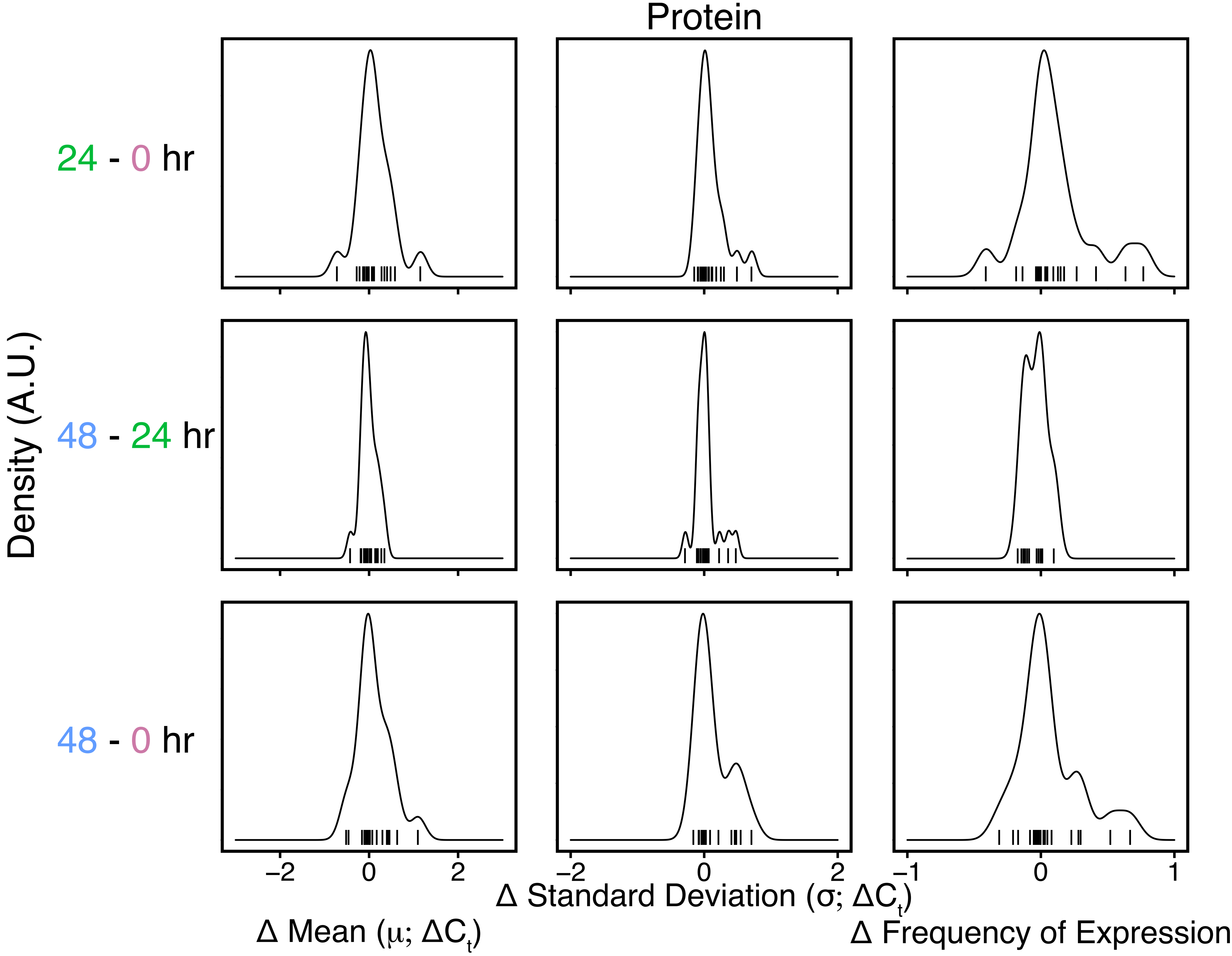

Supplement: Additional file 11: Figure S10. — Change in average, standard deviation, and frequency of expression. Density traces (each with their own arbitrary units) for change in mean, standard deviation, and frequency of expression are shown for genes quantified as both RNA (a) and protein (b). Each row depicts a time point transition (24 – 0 hr, 48 – 24 hr, 48 – 0 hr for the top, middle, and bottom rows, respectively) for every gene with at least two cells above detection in every time point for both RNA and protein (19 genes). The ticks display individual measurements from each time point transition. (PDF 508 kb) [file 13059_2016_1045_MOESM11_ESM.pdf]

FIGURE S11

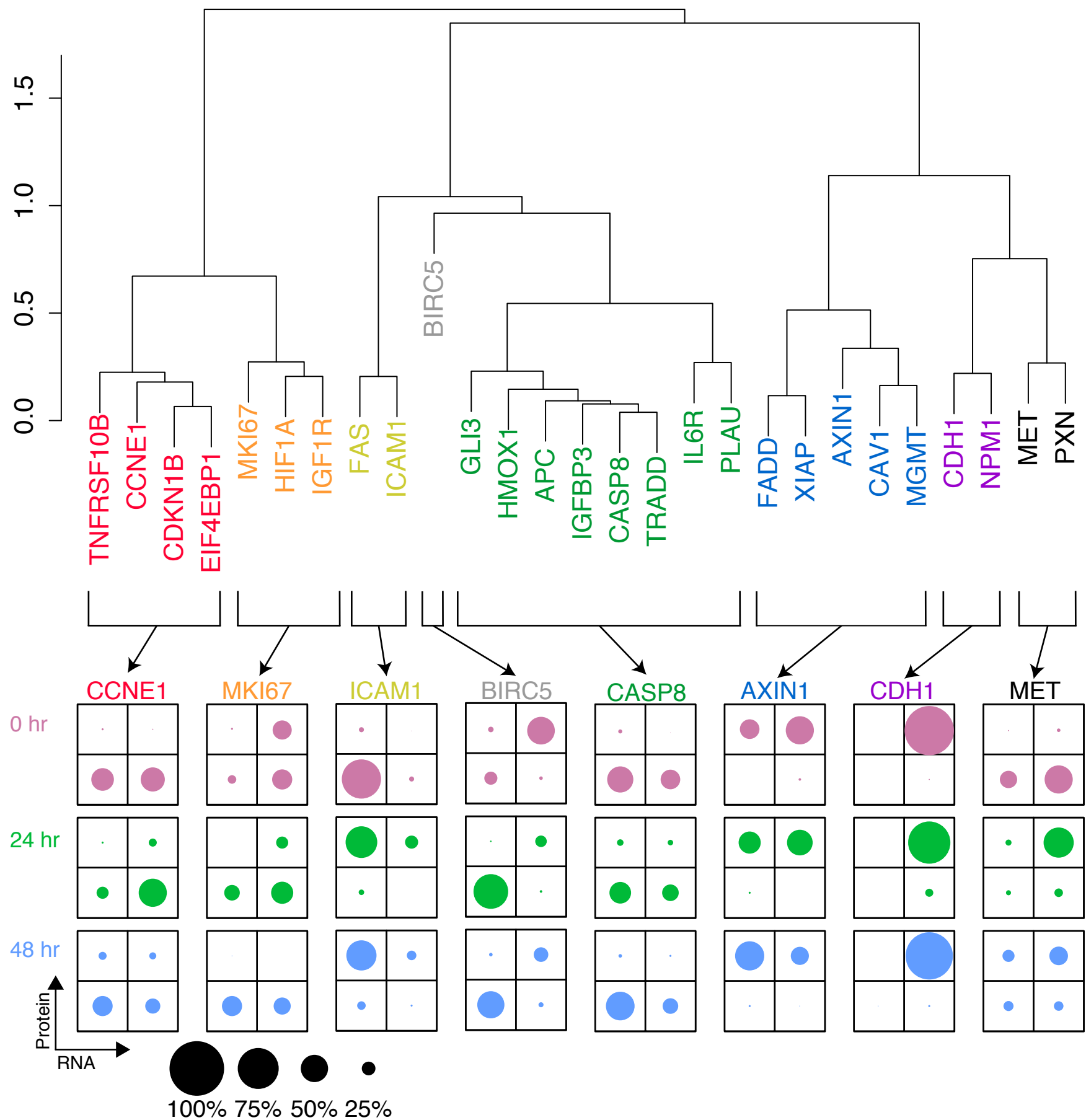

Supplement: Additional file 12: Figure S11. — Analyzing gene trajectories through a time course of PMA stimulation. Genes quantified as both RNA and protein were made binary for each value based on detection or lack thereof. The vector of the proportion of cells in the four quadrants of detected and undetected for RNA and protein across the three stimulation time points were clustered using Spearman correlation. Clusters are divided using a distance metric of 0.75; representative trajectories through time (depicted by color 0 hr = purple, 24 hr = green, 48 hr = blue) are shown for the genes evaluated in situ with the bottom left quadrant representing undetected protein and undetected RNA, the bottom right quadrant representing undetected protein and detected RNA, the top left quadrant representing detected protein and undetected RNA, and the top right quadrant representing detected protein and detected RNA. The size of the dots corresponds to the fraction of cells in that quadrant. (PDF 179 kb) [file 13059_2016_1045_MOESM12_ESM.pdf]

FIGURE S12

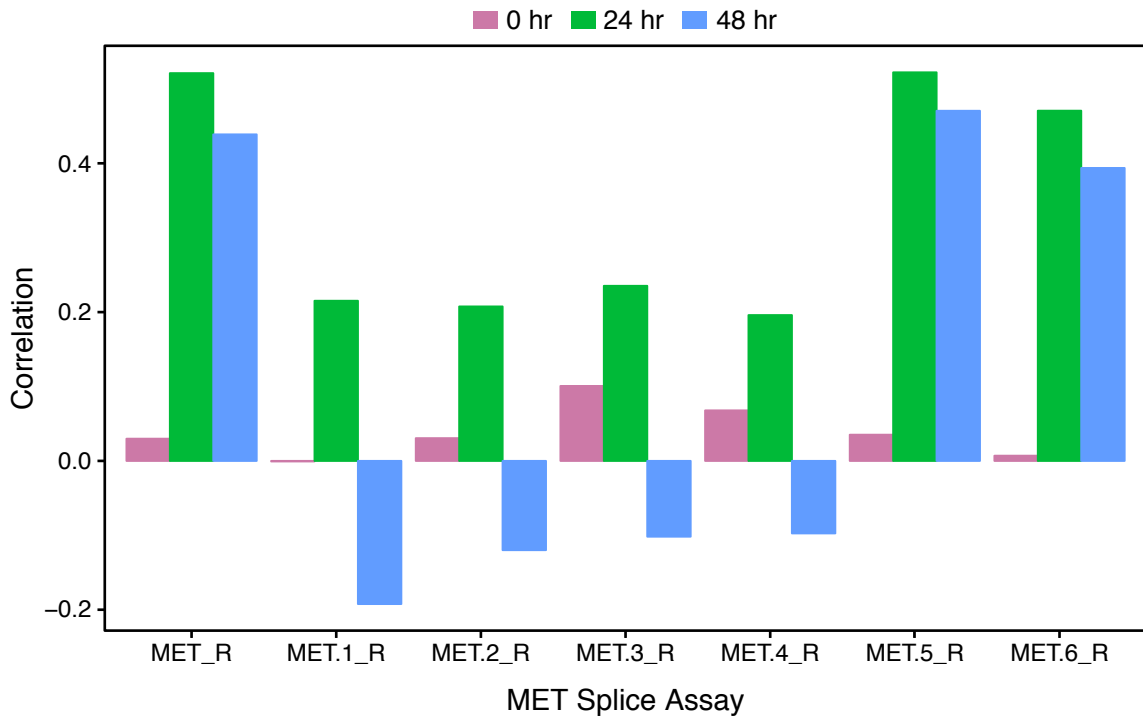

Supplement: Additional file 13: Figure S12. — Correlation between protein and RNA: MET. Presented are the correlations of the various MET STAs with MET protein (by PEA) across time points (0 hr = purple, 24 hr = green, 48 hr = blue). We observe that the strong increase in correlation after stimulation is most noted in MET.5_R (spliced short isoform) and MET.6_R (exon 10), with the other STAs showing a modest increase in correlation at 24 hr that becomes negative by 48 hr. (PDF 122 kb) [file 13059_2016_1045_MOESM13_ESM.pdf]

FIGURE S13

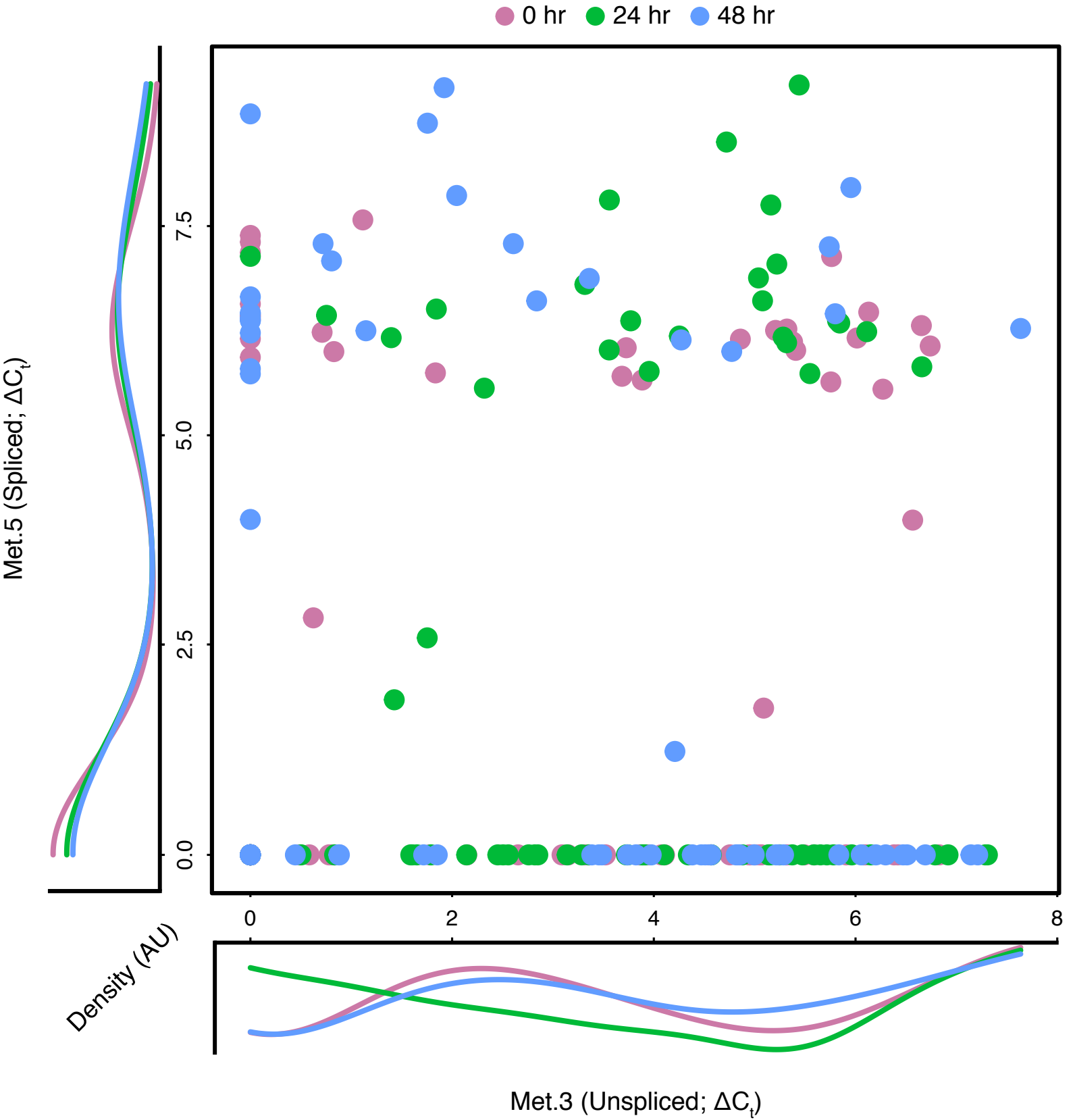

Supplement: Additional file 14: Figure S13. — Spliced vs. unspliced MET transcript. Scatterplot of MET.5_R (spliced, short isoform) vs. MET.3_R (unspliced) with density curves plotted along the axes. Time point indicated by color: 0 hr = purple, 24 hr = green, 48 hr = blue. (PDF 227 kb) [file 13059_2016_1045_MOESM14_ESM.pdf]

FIGURE S14

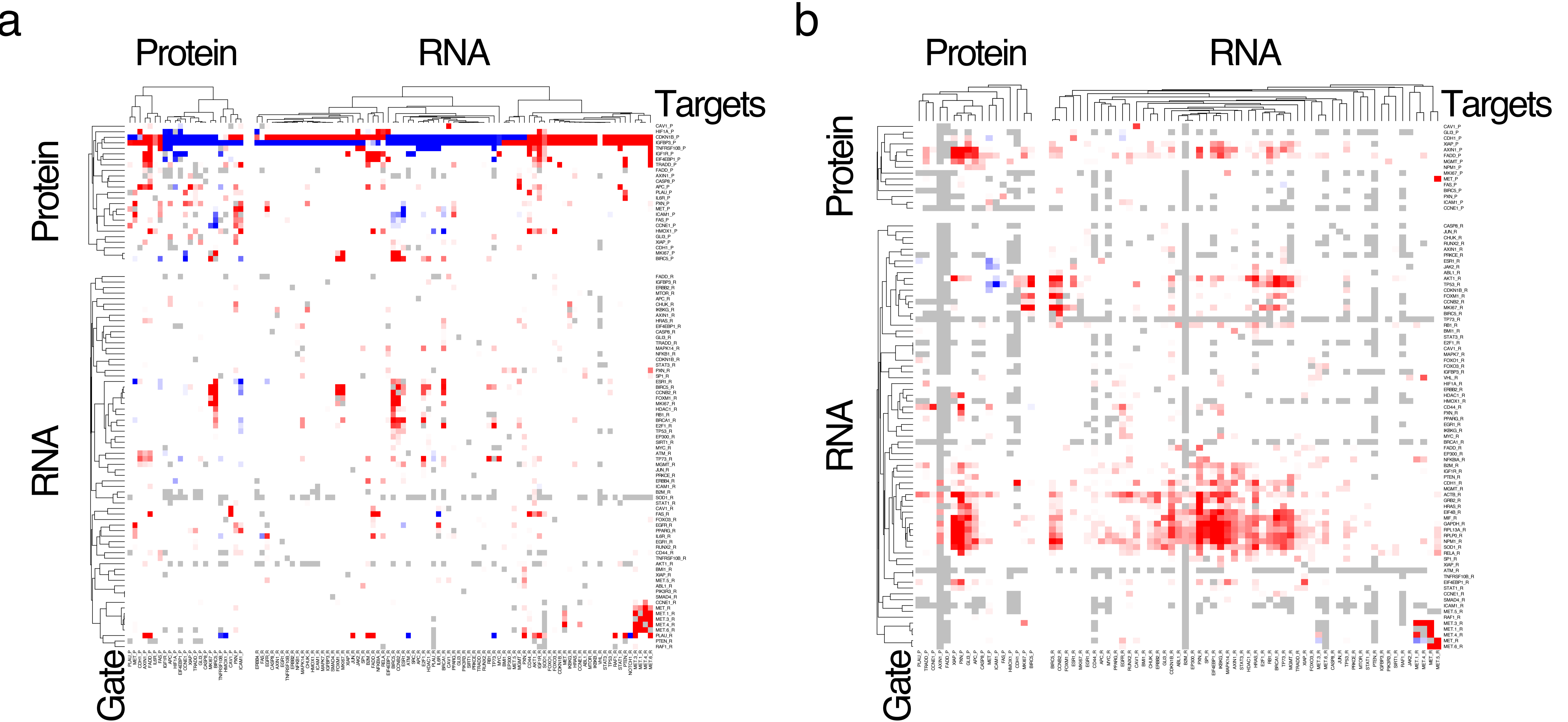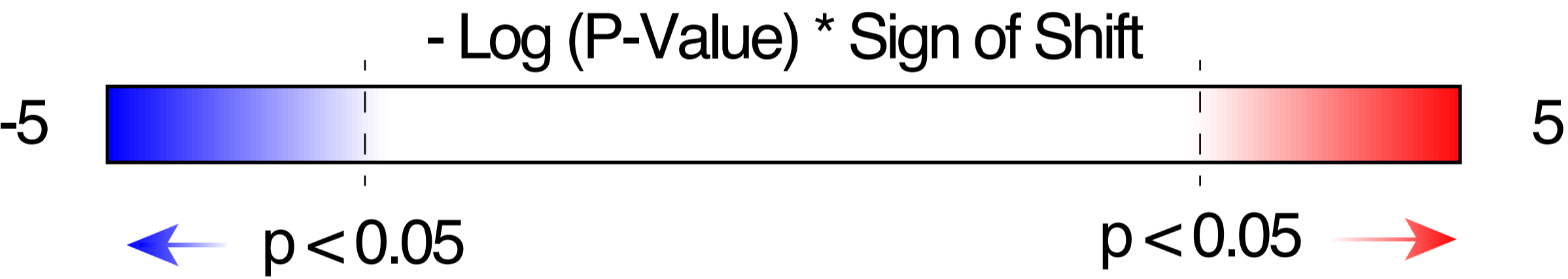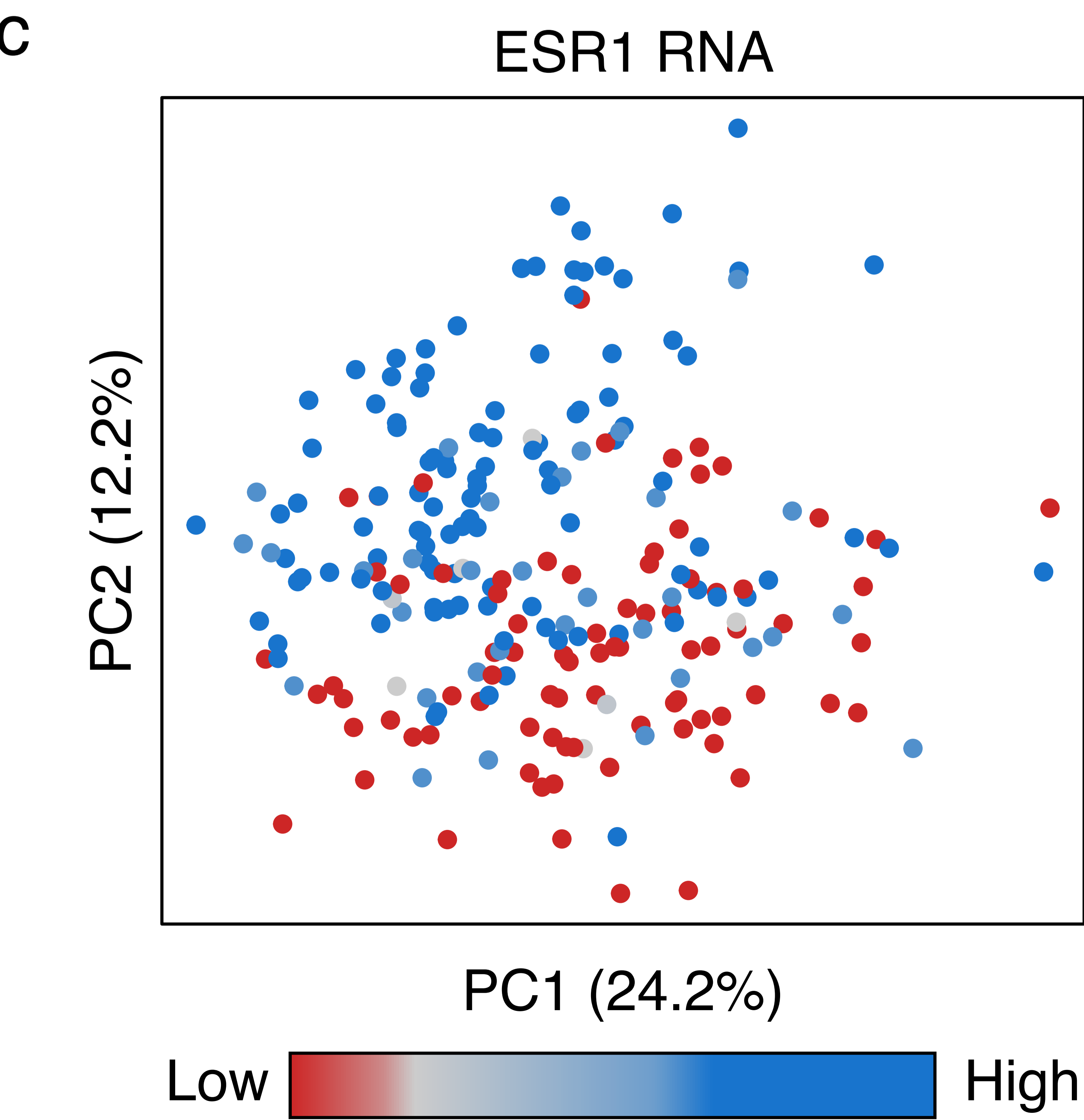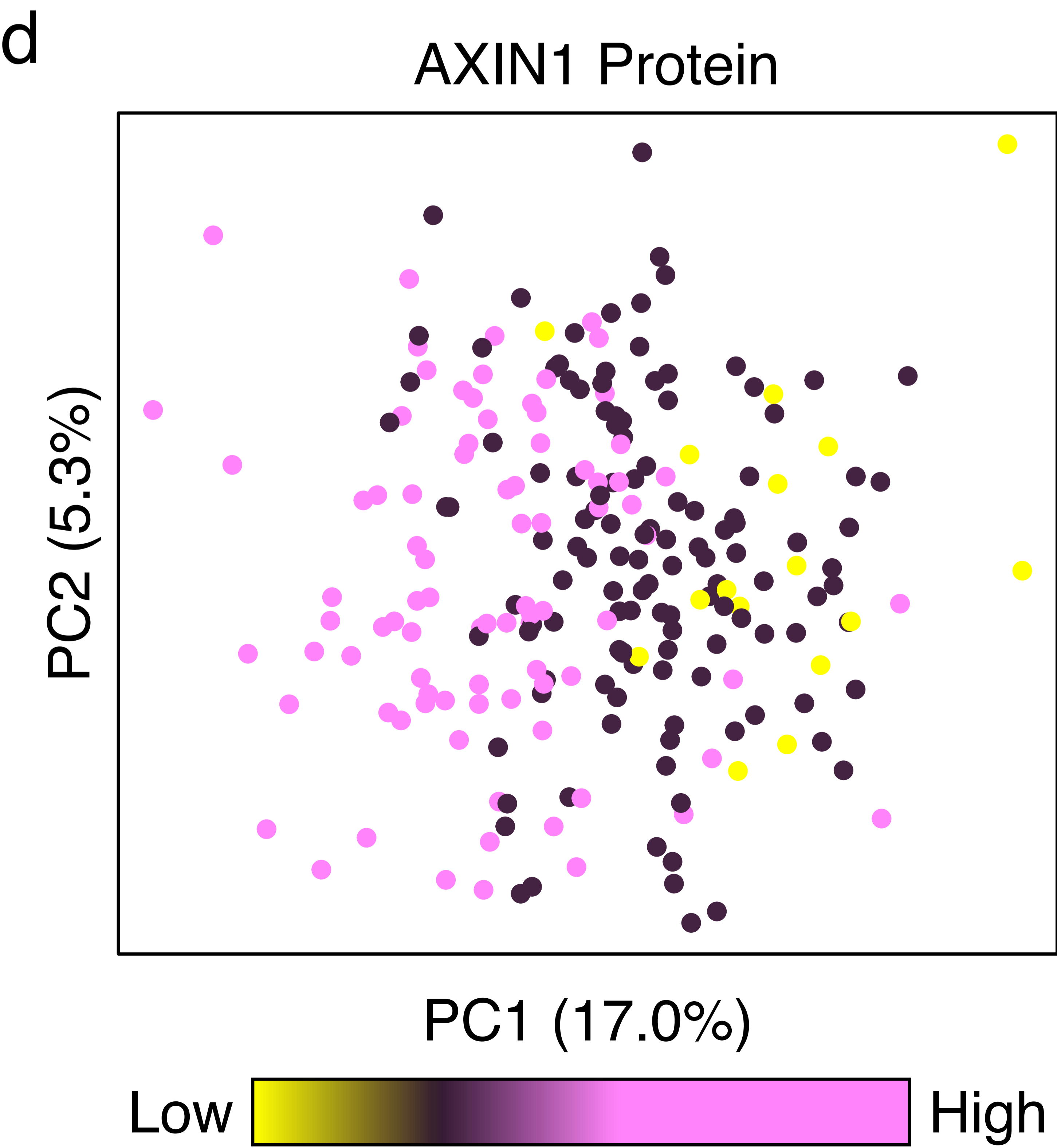

Supplement: Additional file 15: Figure S14. — In silico gating of samples by both discrete and continuous methods. a, b Heatmaps show protein or RNA targets (rows) significantly dysregulated when applying protein or RNA expression gates (columns). Cooler colors indicate a decrease in expressing cells by a Fisher’s exact test (a) or the distribution of expressing cells by a Mann–Whitney U test (b) while warmer colors indicate the inverse. c, d In addition to discrete gating, unsupervised analyses, such as a PCA over protein (c) or RNA levels (d), can also be annotated by additional cellular observations (RNA or protein data, respectively). c ESR1_R expression shows a strong Spearman's ρ of 0.60 with PC2 on a PCA computed from the protein expression. d AXIN1_P expression shows a strong negative ρ of −0.68 with PC1 on a PCA computed over RNA expression. (PDF 964 kb) [file 13059_2016_1045_MOESM15_ESM.pdf]

FIGURE S15

a

Extension Control

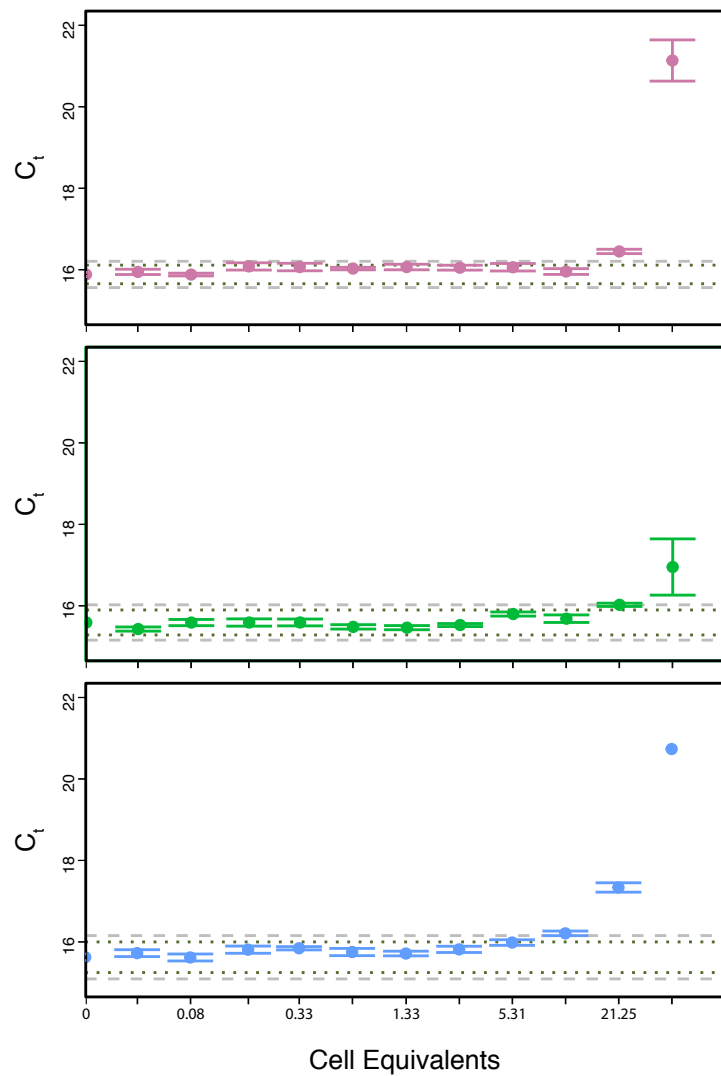

b

Oligo Reference

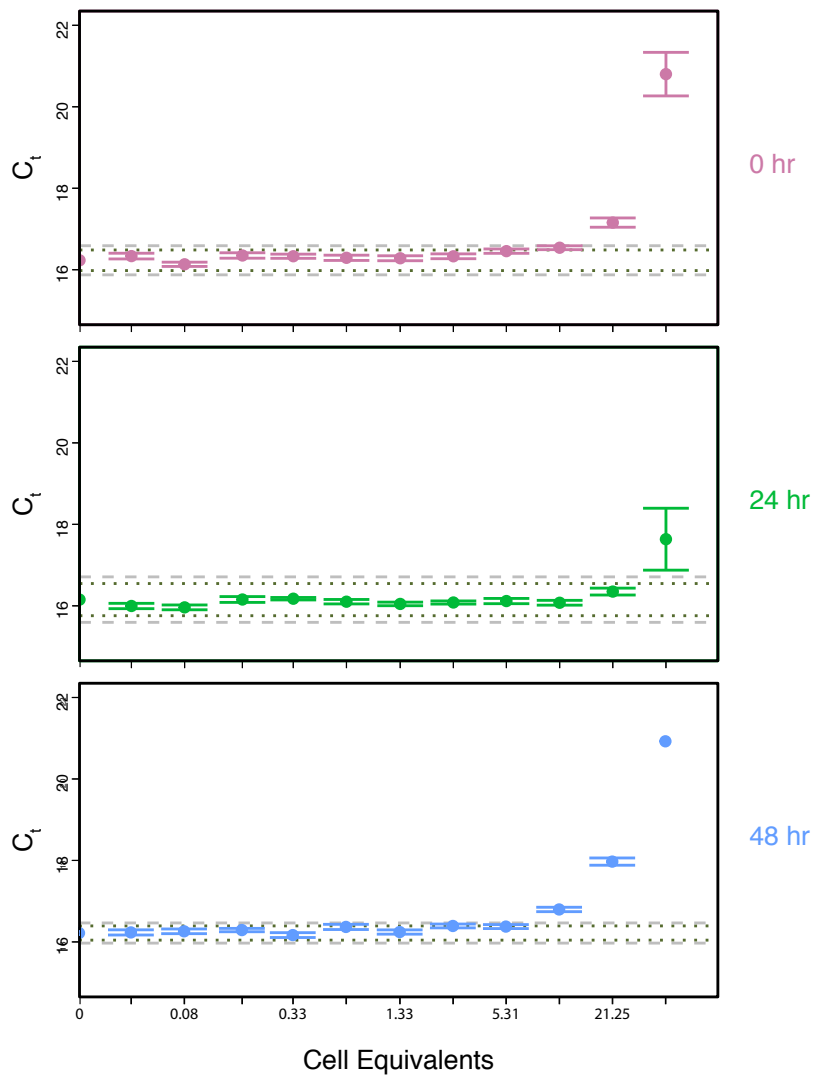

Supplement: Additional file 16: Figure S15. — Analyzing population lysate dilution data. The Ct values observed for the two PEA standards: the extension control (a) and oligo reference (b). Across time points (0 hr = purple, 24 hr = green, 48 hr = blue) and cell dilutions, the deviations from the lysis buffer control are quite small except for the 42.5 and 21.25 cell equivalent dilutions (0.05 and 0.01 probability cutoffs from the background shown in green and gray, respectively). In these deviations are quite small except for the 42.5 and 21.25 cell equivalent dilutions. Therefore, those measurements are excluded from the dilution plots in Additional files 2, 3, and 4: Figures S1, S2, and S3. (PDF 143 kb) [file 13059_2016_1045_MOESM16_ESM.pdf]
